# Comparative genomic analysis of primary tumors and metastases in breast cancer

**DOI:** 10.18632/oncotarget.8349

**Published:** 2016-03-25

**Authors:** François Bertucci, Pascal Finetti, Arnaud Guille, José Adélaïde, Séverine Garnier, Nadine Carbuccia, Audrey Monneur, Emmanuelle Charafe-Jauffret, Anthony Goncalves, Patrice Viens, Daniel Birnbaum, Max Chaffanet

**Affiliations:** ^1^ Département d'Oncologie Moléculaire, Centre de Recherche en Cancérologie de Marseille (CRCM), Institut Paoli-Calmettes, INSERM UMR1068, CNRS UMR725, Marseille, France; ^2^ Département d'Oncologie Médicale, CRCM, Institut Paoli-Calmettes, Marseille, France; ^3^ Faculté de Médecine, Aix-Marseille Université, Marseille, France; ^4^ Département de Biopathologie, CRCM, Institut Paoli-Calmettes, Marseille, France

**Keywords:** array-CGH, breast cancer, genomics, metastasis, sequencing

## Abstract

Personalized medicine uses genomic information for selecting therapy in patients with metastatic cancer. An issue is the optimal tissue source (primary tumor or metastasis) for testing. We compared the DNA copy number and mutational profiles of primary breast cancers and paired metastases from 23 patients using whole-genome array-comparative genomic hybridization and next-generation sequencing of 365 “cancer-associated” genes. Primary tumors and metastases harbored copy number alterations (CNAs) and mutations common in breast cancer and showed concordant profiles. The global concordance regarding CNAs was shown by clustering and correlation matrix, which showed that each metastasis correlated more strongly with its paired tumor than with other samples. Genes with recurrent amplifications in breast cancer showed 100% (*ERBB2*, *FGFR1*), 96% (*CCND1*), and 88% (*MYC*) concordance for the amplified/non-amplified status. Among all samples, 499 mutations were identified, including 39 recurrent (*AKT1*, *ERBB2*, *PIK3CA*, *TP53*) and 460 non-recurrent variants. The tumors/metastases concordance of variants was 75%, higher for recurrent (92%) than for non-recurrent (73%) variants. Further mutational discordance came from very different variant allele frequencies for some variants. We showed that the chosen targeted therapy in two clinical trials of personalized medicine would be concordant in all but one patient (96%) when based on the molecular profiling of tumor and paired metastasis. Our results suggest that the genotyping of primary tumor may be acceptable to guide systemic treatment if the metastatic sample is not obtainable. However, given the rare but potentially relevant divergences for some actionable driver genes, the profiling of metastatic sample is recommended.

## INTRODUCTION

During the last decades, the progression-free survival of patients with metastatic breast cancer has improved [1] thanks to the introduction of targeted therapies such as ERBB2 inhibitors, bevacizumab [2], and mTOR inhibitors [3]. Other promising drugs such as PARP inhibitors [4] or CDK4 inhibitors [5] are in development. However, the benefit is limited to patients’ subgroups that remain to be defined. In this context, the identification of biomarkers of response is fundamental to better tailor these expensive and potentially toxic treatments.

Current personalized medicine is based on the accurate identification of actionable molecular alterations present at the time of relapse. One key issue is whether these must be searched in the metastasis or may be identified in the primary tumor removed some years earlier, but generally available. Metastatic progression is a complex multistep phenomenon characterized by the accumulation of multiple molecular alterations, notably genetic, within cell clones of a primary tumor, oligoclonal and genetically instable [6]. It has thus been suggested that the genomic profile of metastases should be discordant from that of the primary tumor, with the presence of mutations conferring selective growth or invasive advantage to the metastatic cells. New generation sequencing has revealed the genetic heterogeneity between primary and metastases, between metastases, but also within different regions of the same tumor. The analysis of rare subclones within tumors has evidenced the concept of branched evolution where both divergence and independent convergence may happen synchronously in multiple subclonal populations [7]. Such heterogeneity partly explains the emergence of therapeutic resistance, a predominant cause of cancer-related death, and the discrepancies between primary tumor and metastases reported for some clinical biomarkers [8]. In breast cancer, the tumor-metastasis discrepancy rates are 9–18% for estrogen receptor (ER), 24–31% for progesterone receptor (PR), and ~10% for ERBB2 [9]. That led ASCO to recommend, in patients with accessible metastases, the biopsy for diagnostic confirmation and retesting of ER, PR, and ERBB2 status [9].

With the increasing development of targeted therapies and the advent of personalized medicine, the genotyping of metastatic samples, mainly based on array-based comparative genomic hybridization (aCGH) and next-generation sequencing (NGS), is being used in research as molecular screening before enrollment in clinical trials [10, 11], and might enter the routine clinical practice in the coming years. However, obtaining good quality biopsies of metastatic lesions is often challenging: core biopsy specimens tend to be small in size that will not always allow molecular analyses aside from immunohistochemistry (IHC); they tend to be relatively impure because of stromal contamination; rebiopsies are not always possible according to metastatic sites and may be associated with morbidity. Whether the archival specimen of the operated primary tumor accurately contains already the critical genomic alterations present on metastasis is thus a clinically relevant issue, which has been addressed in certain cancers such as colon cancer with discordant results [12, 13].

Here, we have compared the genomic profiles of primary tumors and matched metastasis from 23 patients with breast cancer by using whole-genome high-resolution aCGH and targeted NGS of 365 “cancer-associated” genes. Our aim was to determine the concordance degree of molecular alterations between tumors and their paired metastases, and the possibility of targeted therapy based on the respective genomic profiles.

## RESULTS

### Patients’ characteristics

Twenty-three women with breast cancer were included in the study. Clinicopathological characteristics are summarized in Table 1. Median age at time of breast cancer diagnosis was 41 years (range, 33 to 72). All cases were carcinomas, including different pathological subtypes. As expected, primary tumors showed poor-prognosis features with a high percentage of grade 2–3 (84%), ER-negative (32%), and ERBB2-positive (33%) cases. One tumor/metastasis pair corresponded to synchronous metastasis, and other pairs corresponded to metachronous metastasis. Median delay between the diagnosis of primary cancer and the metastasis profiled was 36 months (range, 0 to 149). The metastasis corresponded either to the first metastatic relapse (*N* = 16) or to the 2nd to 6th metastatic progression (*N* = 7). None of the primary tumors had been exposed to systemic therapy before removal. Regarding the metastatic samples, two patients had not received any systemic therapy before removal or biopsy, whereas 21 had received systemic therapy, mainly chemotherapy and hormone therapy, but also chemotherapy and targeted therapies (anti-ERBB2, and a PI3K inhibitor, BKM120). The main metastatic sites profiled were liver, skin and lymph nodes.

**Table 1:** Clinicopathological characteristics of the 23 patients

Sample ID	Primary Tumor					Systemic treatment between primary tumor and profiled metastasis	Metastatic location	Delay between primary tumor and metastasis (months)	N° of metastatic relapse or progression
	Age at diagnosis	Pathological type	Pathological grade	ER status*	ERBB2 status*				
1	63	Mixed	2	Positive	Negative	Yes (chemoT, hormonoT)	Lymph nodes	26	1
2	52	Ductal	2	Positive	Negative	Yes (chemoT, hormonoT)	Skin	110	2
4	33	Ductal	3	Positive	Negative	Yes (chemoT, hormonoT)	Liver	35	1
5	56	Ductal	3	Negative	Negative	Yes (chemoT)	Lymph nodes	25	1
6	41	Mixed	2	Positive	Positive	Yes (chemoT, hormonoT)	Skin	39	1
7	43	Medullary	3	Negative	Negative	Yes (chemoT)	Muscle	27	1
8	61	Ductal	1	Positive		Yes (chemoT, hormonoT)	Liver	11	1
9	72	Lobular	2	Positive	Negative	Yes (chemoT, hormonoT)	Uterus	61	1
10	41	Ductal	1	Positive	Negative	Yes (chemoT, hormonoT)	Ovary	32	3
11	34	Ductal		Positive	Negative	Yes (chemoT)	Ovary	5	1
12	50	Ductal		Positive		No	Lymph nodes	81	1
13	51	Ductal	3	Negative	Positive	Yes (hormonoT)	Lung	36	1
14	34	Ductal	2	Negative		Yes (chemoT, hormonoT)	Bladder	23	1
15	35	Ductal	1			No	Lymph nodes	88	1
16	38	Ductal	3	Positive	Positive	Yes (chemoT)	Skin	12	1
17	70	Lobular	1	Negative	Negative	Yes (chemoT)	Skin	42	1
18	62	Ductal	3	Negative	Positive	Yes (chemoT, trastuzumab, lapatinib, BKM120, T-DM1)	Skin	25	3
20	60	Ductal	2	Positive	Positive	Yes (chemoT, hormonoT, trastuzumab, lapatinib, T-DM1)	Peritoneum	149	4
21	37	Ductal		Positive	Negative	Yes (chemoT, hormonoT)	Lymph nodes	73	6
22	33	Ductal	3	Positive	Negative	Yes (chemoT, hormonoT, trastuzumab)	Liver	52	4
23	38	Metaplastic	3	Negative	Negative	Yes (chemoT)	Liver	0	1
24	34	Ductal		Positive	Negative	Yes (chemoT, hormonoT)	Liver	63	4
26	38	Ductal	3	Positive	Positive	Yes (chemoT, hormonoT, trastuzumab)	Ovary	78	1

* IHC status: ER (10% positivity cut-off) and ERBB2 (0–3 + score, DAKO HercepTest, with > 1 + defined as positive).

### Copy number profiles

**Figure 1:**
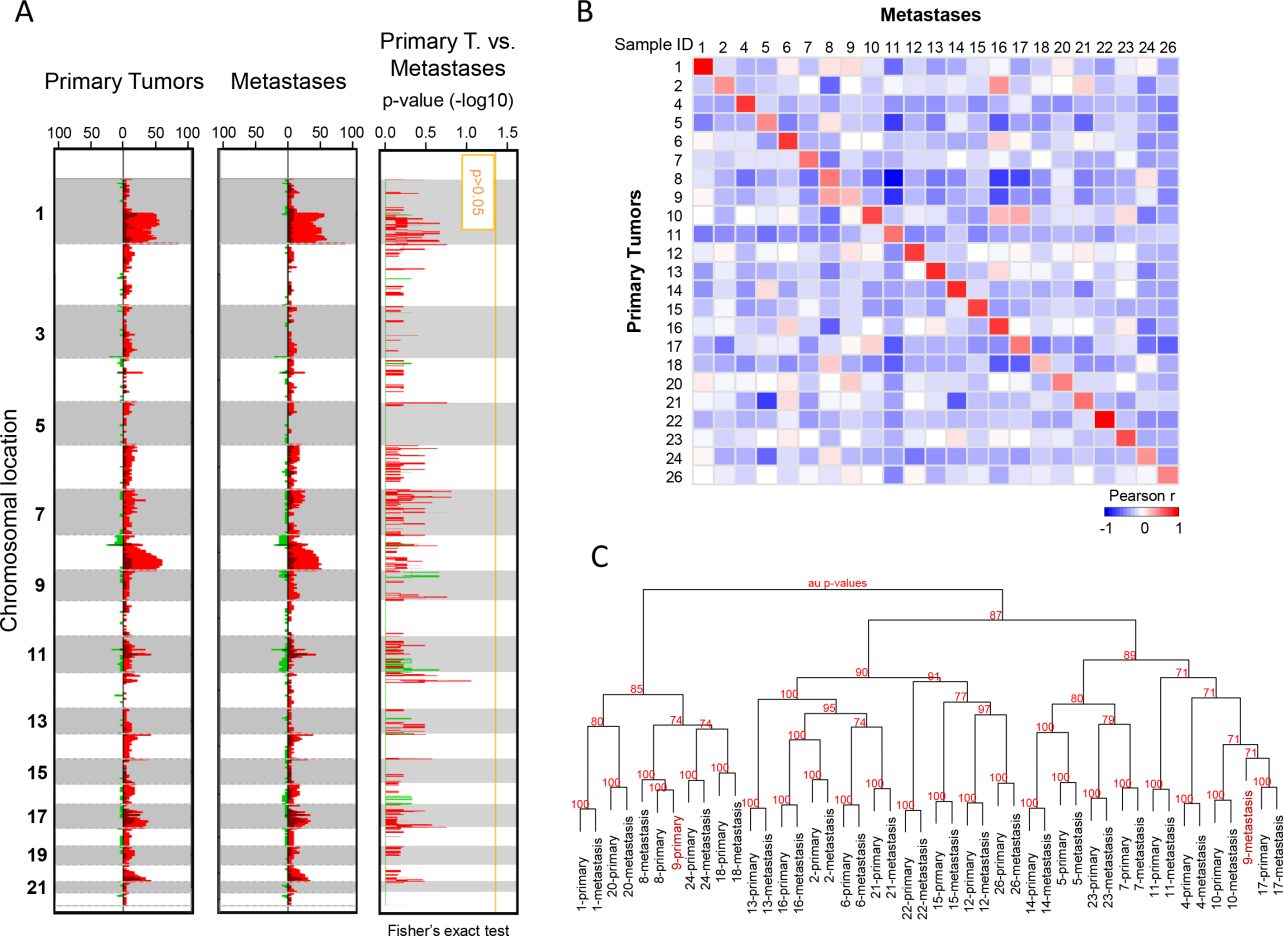
Copy number alteration profiles of primary tumors and metastases (**A**) Frequency plots of genome CNA. Frequencies (horizontal axis, from 0 to 100%) are plotted as a function of chromosome location (from 1 pter to the top, to 22 qter to the bottom), for all primary tumors (*N* = 23) and metastases (*N* = 23). Frequencies of tumors showing CNA are color-coded, with gains in light red, amplifications in dark red, losses in light green, and deletions in dark green. Right: Supervised analysis of CNA frequencies between 23 primary tumors and 23 metastases. Plotted values represent the –log10 *p*-values of the Fisher's exact test, in red for gained/amplified regions and green for lost/deleted regions. The vertical orange line represents the significance threshold. We did not identify any genomic segment significantly differentially altered between primary tumors and metastases. (**B**) Correlation matrix based on the CNA profiles (log2 ratios of all probes) generated between all primary tumors and all metastases: the Pearson coefficient is color-coded according to the scale shown below the matrix. (**C**) Dendrogram of the hierarchical clustering (R-package pvclust) of whole-genome CNAs measured for 46 samples (26 pairs). The AU (Approximately Unbiased) *p*-values provided by multiscale bootstrap resampling indicate the robustness of tumor clusters, larger the *p*-values, more robust the clusters.

We first compared the aCGH genomic profiles of the 23 primary tumors and 23 metastases. Figure 1A (left panel) shows the frequency plots of 23 primary tumors: as expected [14–16], the most frequently gained regions were on 1q, 8q, 11q, 17q and 20q chromosomal arms, whereas the regions frequently lost were on 8p, 11q and 16q. Globally, metastases and primary tumors showed similar altered regions with similar frequencies of alterations, and no region showed a different alteration frequency (Figure 1A). GISTIC analysis confirmed that most of altered regions were similar between primaries and metastases, but a few regions were different such as the *ATM*-including region, which was deleted in metastases (Supplementary Figure 1). The median percentage of probes displaying a CNA per sample was not different between primary tumors (5.18%, range 0.92–33.7%) and metastases (5.83%, range 0.06–23.7%; *p* = 0.846, paired Mann-Whitney test), even if a great variability existed for both types of samples (Supplementary Table 1). As shown by the correlation matrix generated with all probes (Figure 1B), each metastatic sample correlated more strongly with its paired primary tumor than with other samples. Hierarchical clustering of whole DNA copy number data showed that most of paired primary and metastatic samples (22 pairs out of 23) clustered together (Figure 1C), suggesting genetic similarity and potential clonal relationship. For only one pair (patient N°9), samples were distantly related, suggesting distinct genetic relationship.

**Figure 2:**
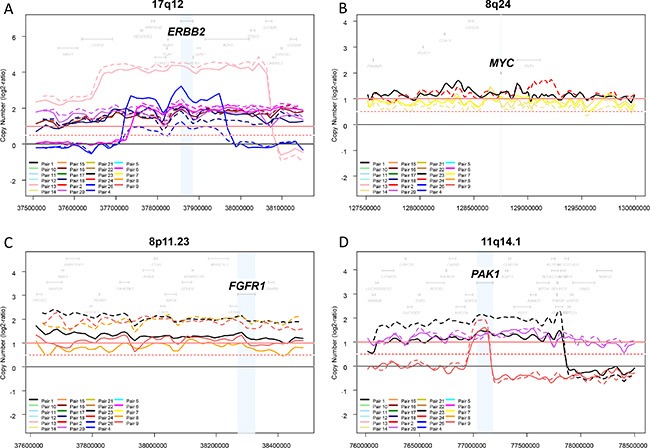
Genomic profiles within four regions frequently amplified in breast cancer The copy number profiles of each region (log2 ratios) were plotted for each of the 46 samples (23 pairs). Different colors correspond to different pairs, and the full line corresponds to the primary tumor and the dashed line to the metastasis. Four regions frequently amplified in breast cancer and one oncogene driver per region are shown: 17q12 and ERBB2 (**A**) 8q24 and MYC (**B**) 8p11.23 and FGFR1 (**C**) and 11q14.1 and PAK1 (**D**).

We then focused the comparison of CNAs on known driver oncogenes located within regions frequently amplified in breast cancer: *ERBB2* (17q12), *CCND1* (11q13.3), *FGFR1* (8p11.23), *MYC* (8q24), and *PAK1* (11q14.1). As expected, *ERBB2* was in our series the most frequently amplified gene, and showed 100% concordance between the aCGH status and the IHC status for the 41 informative samples. The concordance rate of amplified/non-amplified status between primary tumors and paired metastases was 100% for *ERBB2* and *FGFR1,* 96% for *CCND1* and *PAK1*, and 88% for *MYC* (Figure 2), suggesting possible differences regarding some driver genes.

### Mutational profiles

**Figure 3:**
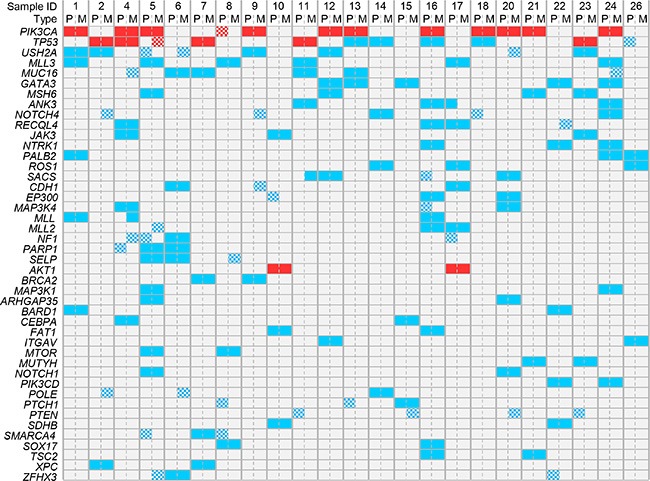
Distribution of mutations in all samples The mutations present in at least 4 out of 46 samples are shown. Genes are ordered from top to bottom by decreasing frequency of mutations. Samples are ordered by patient number. Recurrent mutations are in red and non-recurrent mutations are in blue. The checkerboard pattern indicates the discordant mutations between primary tumors (P) and paired metastases (M).

**Figure 4:**
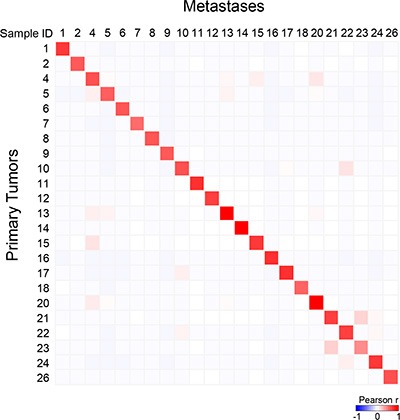
Correlation between each metastatic sample and all primary tumors with respect to mutational profiles Correlation matrix based on the variant allele frequency (VAF) for all detected variants generated between all primary tumors and all metastases: the Pearson coefficient is color-coded according to the scale shown below the matrix.

Among the 365 sequenced genes, 499 mutations, including 414 SNVs (non-synonymous, stop/gain) and 85 indels, were retained as putative somatic alterations within the 46 samples. They corresponded to 298 different mutations (see Supplementary Table 2 for the details of alterations). All samples exhibited at least one mutation. As expected for breast cancers [17], mutational profiles of primary tumors included *AKT1, CDH1, ERBB2, GATA3, MLL3/KMT2C*, *PIK3CA,* and *TP53* mutations (Figure 3). A total of 247 mutations (205 SNVs, 42 indels) were identified among the primary tumors, and 252 (209 SNVs, 43 indels) among the metastases. The median number of mutations per sample was similar between primary tumors (9, range 4 to 24) and metastases (9, range 4 to 29; *p* = 0.709, paired Mann-Whitney test). We measured the similarity between each metastasis and all primary tumors by measuring the correlation of variant allele frequencies (VAF) of all detected variants: each metastasis correlated more strongly with its paired primary tumor than with other samples, suggesting strong similarity (Figure 4). However, the VAF of some variants showed strong differences between primary tumor and metastasis in some cases, such as *PIK3CA* in the pairs N°9 and 24, and *TP53* in the pair N°23 (Supplementary Table 2). This global similarity was also observed in the concordance analysis, as shown by the correlation matrix in Supplementary Figure 2. The global rate of concordance for the detected variants between primary tumor and paired metastases was 75%, with 374 shared variants and 125 unshared variants (Table 2).

**Table 2:** Concordance between primary tumors and paired metastases for all detected variants

Types of mutations	All mutations (*N*)	Unshared mutations (*N*)	Shared mutations (*N*)	Concordance rate
All mutations	499	125	374	75%
Recurrent mutations	39	3	36	92%
Passenger mutations	460	122	338	73%

The 499 variants included 39 recurrent variants (8%; 14 unique) and 460 non-recurrent variants (92%; 284 unique). The 39 recurrent variants (Table 3) concerned four driver genes of breast cancer: *AKT1*, *ERBB2, PIK3CA*, and *TP53*. They were equally distributed with 19 variants in primary tumors and 20 in metastases. Eighteen out of 23 primary tumors harbored at least one of these recurrent variants (two variants in one case) and 17 out of 23 metastases harbored at least one recurrent variant (two variants in three cases) (Supplementary Table 2). The concordance rate (Table 2) was higher for the recurrent variants (92%: 36/39) than for the non-recurrent variants (73%: 338/460; *p* = 0.076, Fisher's exact test, Odds Ratio = 3.1 [CI95 0.93–16.4]). *PIK3CA* variants were the most frequently detected recurrent mutations (23 samples from 12 patients) with one discordant mutation observed in patient N°8, who harbored the E545K variant in the primary tumor but not in the metastasis. *TP53* variants were observed in 11 samples from 6 patients with one discordant mutation observed in patient N°5, who harbored the R273C variant in the metastasis but not in the primary tumor. *AKT1* variants were observed in 2 patients with no discordant mutation. The only *ERBB2* variant (L755S) was observed in patient N°20 in the metastasis but not in the primary tumor. Before the biopsy of the profiled metastasis (4th metastatic progression), this patient had received several lines of anti-ERBB2 drugs (trastuzumab, lapatinib, T-DM1). The L755S *ERBB2* variant induces resistance to lapatinib *via* restriction of kinase conformational flexibility that blocks lapatinib binding [18]. It is likely that the mutation present in this metastasis had been induced by the preceding treatment. Thus, 20 out of 23 tumor/metastasis pairs (87%) were concordant with respect to the detected recurrent variants, whereas 13% were discordant.

**Table 3:** List of 39 recurrent somatic variants detected in the 46 samples

Gene	cDNA mutation	Impact on protein synthesis	Primary tumors occurence (*N*)	Metastases occurence (*N*)
*PIK3CA*	C1616G	P539R	1	1
	G1624A	E542K	1	1
	G1633A	E545K	3	2
	A3140G	H1047R	4	4
	A3140T	H1047L	2	2
	G353A	G118D	1	1
*TP53*	C152G	S51X*	1	1
	G128A	R43H	1	1
	G317A	C106Y	1	1
	T304C	Y102H	1	1
	C421T	R141C	0	1
	G422T	R141L	1	1
*AKT1*	G49A	E17K	2	2
*ERBB2*	T2264C	L755S	0	1
Total			19	20

* Stop codon

### Choice of targeted therapy

Because personalized medicine theoretically relies on the molecular profile of the relapse, we compared for each patient the therapeutic choices guided by the profiling of primary tumor *versus* metastasis using two examples of clinical trials (Supplementary Table 3). Here, the actionable molecular alterations retained for analysis concerned genes coding for molecular targets of drugs proposed in the trials or involved in pathways targeted by these drugs. Furthermore, they had to be activating for oncogenes and biallelic inactivating for tumor suppressor genes such as *PTEN*. First, we focused on the seven drugs proposed in the SHIVA trial [11]: imatinib, everolimus, vemurafenib, sorafenib, erlotinib, dasatinib, and lapatinib combined with trastuzumab. Based on the profiling of metastasis, 15 patients (65%) would have been candidate to at least one targeted therapy (everolimus: 13; lapatinib plus trastuzumab: 2), whereas 16 (70%) would have been candidate (everolimus: 14; lapatinib plus trastuzumab: 2) according to the profiling of primary tumor. The concordance degree with respect to therapeutic selection was 96% (22 out of 23). Then, we focused on the five drugs proposed in the ongoing MOST trial (NCT02029001): everolimus, nilotinib, sorafenib, pazopanib, and lapatinib. The results were similar, with a concordance rate of therapeutic selection of 96%, with 17 patients (74%) candidate to at least one targeted therapy (everolimus: 14, lapatinib: 2, nilotinib: 1) according to the molecular profile of the metastasis, *versus* 18 (78%: 15 for everolimus, 2 for lapatinib, 1 for nilotinib) according to the profile of primary tumor. Thus, in both trials, the metastasis profiling did not reveal additional actionable therapeutic target as compared with the profiling of primary tumor.

## DISCUSSION

Our objective was to assess the concordance of high-throughput CNA and mutational profiles between primary breast cancers and paired metastases. We showed in our series of 23 patients that overall those profiles are concordant and the resulting selection of targeted therapy would be the same in all but one patient. However, some differences that might be extremely relevant were identified for some genes recurrently altered in breast cancer.

Our analysis was based on whole-genome high-resolution aCGH and targeted NGS of 365 genes chosen for their relevance in oncology by biologists and medical oncologists of our institution. We are currently using these technologies in the PERMED trial (NCT02342158) that aims to establish the genomic profile of advanced tumors as pre-therapeutic screening tool. All samples; primary tumors and metastases, harbored many CNAs and mutations commonly found in breast cancer [17]. The profiled metastatic samples corresponded to different metastatic locations and timings. Different combinations of chemotherapy, hormone therapy and anti-ERBB2 therapies had been delivered between the removals of the primary tumor and the paired metastasis. In term of genes tested, genomic data of paired samples may be compared in several ways. Our analysis was both global, integrating all genes together, but also individual, concerning genes with known oncogenic and recurrent alterations in breast cancer and for which targeted therapies are available.

Despite the above-quoted divergences and the known genetic instability of cancer cells, we found a very high level of global concordance between primary and secondary tumors. The concordance with respect to whole CNAs was first suggested by similar frequency plots and numbers of alterations per sample, then more importantly by clustering and correlation matrix which showed that each metastasis correlated more strongly with its paired primary tumor than with other samples. At the gene levels, genes with recurrent amplifications in breast cancer showed different degrees of concordance: 100% for *ERBB2* and *FGFR1,* 96% for *CCND1,* but 88% for *MYC,* suggesting possible differences for driver genes. The assessment of global similarity of mutational profiles was based on different criteria: number of mutations *per* sample, correlation matrix, and concordance analysis of detected variants. This later was 75% when we considered all variants, but higher for recurrent variants (92%), which concern driver genes involved in disease progression, than for non-recurrent variants (73%), which are generally random/passenger and the consequence of genomic instability. Of course, our findings need to be interpreted in the context of clinically relevant clonality: here we chose a 2% cut-off, but the optimal clonal frequency is currently unknown with different values used ranging between 2 and 10%. Reanalysis of our data using 5% and 10% cut-offs decreased the degree of concordance with respective rates of 70 and 60% for all variants, 82 and 77% for recurrent variants, and 69 and 59% for non-recurrent variants (data not shown). The three recurrent variants for which we found rare cases of divergent mutation (*TP53, PIK3CA,* and *ERBB2*) are part of a list of 16 genes already reported with frequent clonal divergence in a list of 46 genes tested [19]. Their discordance between primary tumor and metastasis may have several explanations: false-negativity due to low cellularity (but cellularity in our cohort was not different between the paired samples) or intra-sample heterogeneity, or true negativity: the two cases with novel mutation in the metastasis (*TP53, ERBB2*) may indicate accumulation of mutations over time associated with the therapeutic resistance (due to previous lapatinib treatment for *ERBB2*), whereas the case with novel mutation in the primary tumor (*PIK3CA*) may suggest that the metastasis branched off before the acquisition of this mutation within the primary tumor. Further degree of mutational discordance came from very different VAF for some variants between primaries and metastases, differences that might have important clinical implications, notably for treatment.

Some comparative studies based on high-throughput molecular analyses have been published and reported similar high degree of global concordance. In breast cancer, at least five studies are available. In a series of 22 matched primary-recurrences sequenced using targeted NGS (196-gene panel), the concordance rate was 85% for the known driver gene mutations [20]. Similarly, high-resolution aCGH profiling of 20 pairs showed strong concordance between the primary tumors and paired lymph node metastases, suggesting high clonal relationship [21]. In a series including 79 primary/metastasis pairs sequenced using targeted NGS (46-gene panel), the concordance rate for all detected variants was 84% [19]. Very good concordance (100% for CNAs, 80% for mutations) was observed for the two paired primary-metastases profiled using whole-genome aCGH and whole-exome, whereas the four bilateral breast cancers pairs showed discordant profiles [22]. Finally, no significant difference was observed between primary tumor and brain metastasis in a series of 15 pairs profiled using targeted NGS of 50 genes [23]. Strong concordance was also observed between the transcriptional profiles of primary tumors and paired metastases [24, 25]. High mutational concordance (94%) between tumors and metastases profiled using targeted NGS has been reported for known recurrent genomic alterations in a series of 15 pairs of non-small cell lung cancers [26]. In colon cancer profiled using targeted NGS, the concordance rates were 78% (90% for recurrent variants), 79%, and 85% in respective series of 13 pairs [27], 69 pairs [28], and 86 pairs [19]. By contrast, discordance in CNA of potential clinical relevance was reported in urothelial carcinoma [29].

Finally, using two examples of clinical trials of personalized medicine, we showed that the chosen targeted therapy would have been strongly concordant (96%) between two choice based on the profiling of primary tumor and paired metastasis respectively. Of note, the proportion of patients candidate to one of the proposed targeted therapies was very similar between the two trials, ranging from 65 to 74%, as previously reported [10, 11, 20, 30]. Of course, our result remains dependent on the list of drugs tested. For example, the *NOTCH4* mutation observed in the metastasis from patient N°9 but not her primary tumor might impact in a near future the therapeutic decision, but no NOTCH4 inhibitor was included in the list of drugs tested here.

To our knowledge, this study is the first one, which combines both whole-genome aCGH and targeted NGS of a panel of more than 300 genes to breast cancer pairs, and which compares the therapeutic selection based on the profiles of primary tumors and metastases. Of course, it displays some limitations: i) the small number of cases - even if it is the second largest study after that recently reported by the MD Anderson team [19]-, that should enlarge in the future thanks to the recently launched PERMED trial, provided that the frozen primary tumor is available for profiling; ii) the heterogeneity of the population in terms of molecular subtypes, locations and timings of metastases, even if the concordance does not seem different according to these parameters, but the number of pairs precluded any statistical analysis; iii) the delivery of different systemic treatments before the metastatic progression; iv) the relatively small number of genes analyzed by NGS, even if more comprehensive sequencing (whole exome, whole genome) did not identify any additional metastasis-specific actionable alterations in small recent series of breast [22] and colon [28] cancers, when compared to targeted NGS; v) the relatively small number of drugs available in the two tested clinical trials of personalized medicine, when compared to the much higher number of therapeutic targets tested. Whether the concordance rate in the therapeutic decision would be as high as 96% with drugs targeting all screened genes remains unknown, even if we showed strong concordance of recurrent alterations between primaries and metastases. Finally, the comparison between primaries and metastases should not be limited to CNA and mutational profiles, but could include notably proteomics and phosphoproteomics analyses, as well as preclinical models comparing the predictive effect for drug sensitivity of molecular alterations found in primaries and metastases. But today, aCGH and NGS represent the backbone of personalized medicine.

In conclusion, we have evidenced a high level of global concordance, but also a small but actual degree of quali- and/or quantitative divergence for some actionable driver genes. This is an important information for future studies of personalized medicine in metastatic patients. Indeed, because the addition or loss of one single mutation may be extremely relevant by profoundly affecting the signal transduction machinery, such studies should be designed on the basis of genomic profiling of contemporary sample; this is in agreement with the current ASCO guidelines, which recommend the biopsy for retesting ER, PR, and ERBB2 in patients with accessible metastases [9]. However, since the genotyping of the primary tumor seems sufficient to guide systemic treatment in the vast majority of cases, it is acceptable in cases where metastatic location, patient or doctor preference, comorbidity or cost make procurement of a more contemporary specimen untenable. Finally, the profiling of metastatic samples will be crucial not only to help understand the metastatic process and the resistance mechanisms by identifying the molecular alterations found in concordant *versus* divergent primary and metastatic tumor pairs [7], but also to collect precious data for future research.

## MATERIALS AND METHODS

### Breast cancer samples

Potential patients were retrospectively searched in our institutional breast cancer database. Inclusion criteria were: women, invasive breast carcinoma treated at the Institut Paoli-Calmettes, available frozen samples of both primary tumor and paired metastasis, tumor cellularity of at least 50% as assessed by one pathologist (ECJ) on tumor sections before DNA extraction, available clinicopathological data, and written informed patient's consent. The study was approved by our institutional “Comité d'Orientation Stratégique” (N°13-002). Forty-six tissue samples from 23 patients were identified. Samples had been collected by surgery or imaging-guided biopsies and macrodissected and frozen in liquid nitrogen within 30 minutes of removal. Tumor DNA was extracted as previously described [31]. Quality was controlled on polyacrylamide gel electrophoresis, and concentration assessed by using Qubit dsDNA BR Assay.

### Array-comparative genomic hybridization

DNA copy number alterations (CNA) were determined by using high-resolution CGH microarrays (SurePrint G3 Human 4 × 180, Agilent, France) as previously described [31]. Tumor DNA was cohybridized with a pool of 13 normal male DNA as reference. Scanning was done with Agilent Autofocus Dynamic Scanner (G2565BA, Agilent). Data analysis and visualization were done with CGH Analytics 3.4 software (Agilent). Data extraction (log_2_ ratio) was done from CGH analytics, while normalized and filtered log_2_ ratio were obtained from “Feature extraction” software (Agilent). We eliminated data generated by probes mapped to X and Y chromosomes. The final dataset included 161,068 unique probes covering 16,684 genes and intergenic regions according to the hg19/NCBI human genome mapping database (build 37).

Data were analyzed using circular binary segmentation as implemented in the DNA copy R/Bioconductor package [32] with default parameters to translate intensity measurements in regions of equal copy number, each region being defined by at least five consecutive probes. Thus, each probe was assigned a segment value referred to as its “smoothed” value. We used two different threshold values (log_2_ ratio > |0.5|, and |1|) to distinguish low (gain/loss) from high (amplification/deletion) level CNAs respectively [31]. To identify altered regions, we used the GISTIC 2.0 (v2.0.21) algorithm [33], which computes for each segment through the genome a score based on the frequency of CNA combined with its amplitude, with bootstrapping to calculate the significance level (*q* < 0.25).

### Next-generation sequencing

Targeted NGS was applied to a custom-made panel of 365 “cancer-associated” genes selected for their involvement in cancers (CCP-V6 panel; Supplementary Table 4). For each sample, we prepared the DNA libraries of all coding exons and intron-exon boundaries of all genes using the HaloPlex Target Enrichment System (Agilent, Santa Clara, CA, USA) as described [34]. Sequencing was done using the 2 × 150-bp paired-end technology on the Illumina MiSeq platform according to the manufacturer's instructions (Illumina, San Diego, CA, USA).

Sequence data were aligned to the human reference genome (UCSC hg19) using Burrows-Wheeler Aligner [35]. Samples were sequenced at an average depth of 300× for the targeted regions. Bam files were processed as described [34]. Then, the single nucleotide variants (SNVs) calling was done with FreeBayes version 0.9.9 [36] with a minimal alternate variant frequency and coverage set at 0.02 and 10. Insertions/deletions (indels) calling was done using GATK haplotype caller version 2.5-2-gf57256b [37] with default parameters. The variants, i.e SNVs and indels, were annotated with the Annotate Variation Software (ANNOVAR, version 2013-11-12). Known variants found in dbsnp129 and dbsnp137 with a variant allele frequency (VAF) superior to 1% (1000 g or ESP6500) were removed. Finally, low frequency SNVs and indels that were suspected to be false positive were systematically inspected with IGV version 2.3.32 [38, 39].

### Statistical analysis

The frequency of CNAs, computed for each probe locus, was compared between tumors and metastases using the Fisher's exact test. The percentage of probes displaying a CNA per sample was calculated as the total number of probes with CNAs divided by the total number of probes. We analyzed the correlation (Pearson coefficient) of CNA profiles (log_2_ ratio of all probes) of each metastasis with all primary tumors. Hierarchical clustering of whole-genome copy number data was also applied to assess the global genetic similarity between the primaries and metastases: we used the R-package pvclust [40] with the following parameters: Ward's agglomerative method, Pearson correlation and 100 bootstrap replications to assess the robustness of clusters.

Regarding the variants, the similarity of samples was measured using the Pearson correlation based on the VAF for all detected variants of each metastasis with all primary tumors. Concordance analysis was done as described [22]. Recurrent variants were defined as alterations present in 10 or more samples in COSMIC V68; other variants were defined as non-recurrent. Correlations between sample groups and variables were calculated with the Fisher's exact test (qualitative variables), and the Mann-Whitney test (continuous variables). We also compared for each patient the selection of targeted therapies guided by the profiling of primary tumor *versus* metastasis by focusing our analysis on genes coding for molecular targets of drugs proposed in the SHIVA [11] and MOST (NCT02029001) trials. The choice of therapy was based on the guidelines proposed in each respective protocol. All statistical tests were two-sided at the 5% level of significance. Analyses were done in the R software (version 2.15.2).

## SUPPLEMENTARY MATERIALS FIGURES AND TABLES



## References

[1] Giordano SH, Temin S, Kirshner JJ, Chandarlapaty S, Crews JR, Davidson NE, Esteva FJ, Gonzalez-Angulo AM, Krop I, Levinson J, Lin NU, Modi S, Patt DA (2014). Systemic therapy for patients with advanced human epidermal growth factor receptor 2-positive breast cancer: American Society of Clinical Oncology clinical practice guideline. J Clin Oncol.

[2] Miller K, Wang M, Gralow J, Dickler M, Cobleigh M, Perez EA, Shenkier T, Cella D, Davidson NE (2007). Paclitaxel plus bevacizumab versus paclitaxel alone for metastatic breast cancer. N Engl J Med.

[3] Baselga J, Campone M, Piccart M, Burris HA, Rugo HS, Sahmoud T, Noguchi S, Gnant M, Pritchard KI, Lebrun F, Beck JT, Ito Y, Yardley D (2012). Everolimus in postmenopausal hormone-receptor-positive advanced breast cancer. N Engl J Med.

[4] Gelmon KA, Tischkowitz M, Mackay H, Swenerton K, Robidoux A, Tonkin K, Hirte H, Huntsman D, Clemons M, Gilks B, Yerushalmi R, Macpherson E, Carmichael J (2011). Olaparib in patients with recurrent high-grade serous or poorly differentiated ovarian carcinoma or triple-negative breast cancer: a phase 2, multicentre, open-label, non-randomised study. Lancet Oncol.

[5] Turner NC, Ro J, Andre F, Loi S, Verma S, Iwata H, Harbeck N, Loibl S, Huang Bartlett C, Zhang K, Giorgetti C, Randolph S, Koehler M (2015). Palbociclib in Hormone-Receptor-Positive Advanced Breast Cancer. N Engl J Med.

[6] Hanahan D, Weinberg RA (2011). Hallmarks of cancer: the next generation. Cell.

[7] Gerlinger M, Rowan AJ, Horswell S, Larkin J, Endesfelder D, Gronroos E, Martinez P, Matthews N, Stewart A, Tarpey P, Varela I, Phillimore B, Begum S (2012). Intratumor heterogeneity and branched evolution revealed by multiregion sequencing. N Engl J Med.

[8] Vignot S, Besse B, Andre F, Spano JP, Soria JC (2012). Discrepancies between primary tumor and metastasis: a literature review on clinically established biomarkers. Crit Rev Oncol Hematol.

[9] Van Poznak C, Somerfield MR, Bast RC, Cristofanilli M, Goetz MP, Gonzalez-Angulo AM, Hicks DG, Hill EG, Liu MC, Lucas W, Mayer IA, Mennel RG, Symmans WF (2015). Use of Biomarkers to Guide Decisions on Systemic Therapy for Women With Metastatic Breast Cancer: American Society of Clinical Oncology Clinical Practice Guideline. J Clin Oncol.

[10] Andre F, Bachelot T, Commo F, Campone M, Arnedos M, Dieras V, Lacroix-Triki M, Lacroix L, Cohen P, Gentien D, Adelaide J, Dalenc F, Goncalves A (2014). Comparative genomic hybridisation array and DNA sequencing to direct treatment of metastatic breast cancer: a multicentre, prospective trial (SAFIR01/UNICANCER). Lancet Oncol.

[11] Le Tourneau C, Delord JP, Goncalves A, Gavoille C, Dubot C, Isambert N, Campone M, Tredan O, Massiani MA, Mauborgne C, Armanet S, Servant N, Bieche I (2015). Molecularly targeted therapy based on tumour molecular profiling versus conventional therapy for advanced cancer (SHIVA): a multicentre, open-label, proof-of-concept, randomised, controlled phase 2 trial. Lancet Oncol.

[12] Vermaat JS, Nijman IJ, Koudijs MJ, Gerritse FL, Scherer SJ, Mokry M, Roessingh WM, Lansu N, de Bruijn E, van Hillegersberg R, van Diest PJ, Cuppen E, Voest EE (2012). Primary colorectal cancers and their subsequent hepatic metastases are genetically different: implications for selection of patients for targeted treatment. Clin Cancer Res.

[13] Vakiani E, Janakiraman M, Shen R, Sinha R, Zeng Z, Shia J, Cercek A, Kemeny N, D'Angelica M, Viale A, Heguy A, Paty P, Chan TA (2012). Comparative genomic analysis of primary versus metastatic colorectal carcinomas. J Clin Oncol.

[14] Andre F, Job B, Dessen P, Tordai A, Michiels S, Liedtke C, Richon C, Yan K, Wang B, Vassal G, Delaloge S, Hortobagyi GN, Symmans WF (2009). Molecular characterization of breast cancer with high-resolution oligonucleotide comparative genomic hybridization array. Clin Cancer Res.

[15] van Beers EH, Nederlof PM (2006). Array-CGH and breast cancer. Breast Cancer Res.

[16] Bekhouche I, Finetti P, Adelaide J, Ferrari A, Tarpin C, Charafe-Jauffret E, Charpin C, Houvenaeghel G, Jacquemier J, Bidaut G, Birnbaum D, Viens P, Chaffanet M (2011). High-resolution comparative genomic hybridization of inflammatory breast cancer and identification of candidate genes. PLoS One.

[17] Cancer Genome Atlas N (2012). Comprehensive molecular portraits of human breast tumours. Nature.

[18] Trowe T, Boukouvala S, Calkins K, Cutler RE, Fong R, Funke R, Gendreau SB, Kim YD, Miller N, Woolfrey JR, Vysotskaia V, Yang JP, Gerritsen ME (2008). EXEL-7647 inhibits mutant forms of ErbB2 associated with lapatinib resistance and neoplastic transformation. Clin Cancer Res.

[19] Goswami RS, Patel KP, Singh RR, Meric-Bernstam F, Kopetz ES, Subbiah V, Alvarez RH, Davies MA, Jabbar KJ, Roy-Chowdhuri S, Lazar AJ, Medeiros LJ, Broaddus RR (2015). Hotspot mutation panel testing reveals clonal evolution in a study of 265 paired primary and metastatic tumors. Clin Cancer Res.

[20] Meric-Bernstam F, Frampton G, Ferrer-Lozano J, Yelensky R, Palmer GA, Cronin MT, Stephens PJ, Stemke Hale K, Barrera J, Burgues O, Lluch A, Mills GB, Gonzalez-Angulo AM (2012). Cancer gene profile of metastatic breast cancer. J Clin Oncol.

[21] Vollebergh MA, Klijn C, Schouten PC, Wesseling J, Israeli D, Ylstra B, Wessels LF, Jonkers J, Linn SC (2014). Lack of genomic heterogeneity at high-resolution aCGH between primary breast cancers and their paired lymph node metastases. PLoS One.

[22] Song F, Li X, Song F, Zhao Y, Li H, Zheng H, Gao Z, Wang J, Zhang W, Chen K (2015). Comparative genomic analysis reveals bilateral breast cancers are genetically independent. Oncotarget.

[23] Lee JY, Park K, Lim SH, Kim HS, Yoo KH, Jung KS, Song HN, Hong M, Do IG, Ahn T, Lee SK, Bae SY, Kim SW (2015). Mutational profiling of brain metastasis from breast cancer: matched pair analysis of targeted sequencing between brain metastasis and primary breast cancer. Oncotarget.

[24] Perou CM, Sorlie T, Eisen MB, van de Rijn M, Jeffrey SS, Rees CA, Pollack JR, Ross DT, Johnsen H, Akslen LA, Fluge O, Pergamenschikov A, Williams C (2000). Molecular portraits of human breast tumours. Nature.

[25] Weigelt B, Hu Z, He X, Livasy C, Carey LA, Ewend MG, Glas AM, Perou CM, Van't Veer LJ (2005). Molecular portraits and 70-gene prognosis signature are preserved throughout the metastatic process of breast cancer. Cancer Res.

[26] Vignot S, Frampton GM, Soria JC, Yelensky R, Commo F, Brambilla C, Palmer G, Moro-Sibilot D, Ross JS, Cronin MT, Andre F, Stephens PJ, Lazar V (2013). Next-generation sequencing reveals high concordance of recurrent somatic alterations between primary tumor and metastases from patients with non-small-cell lung cancer. J Clin Oncol.

[27] Vignot S, Lefebvre C, Frampton GM, Meurice G, Yelensky R, Palmer G, Capron F, Lazar V, Hannoun L, Miller VA, Andre F, Stephens PJ, Soria JC (2015). Comparative analysis of primary tumour and matched metastases in colorectal cancer patients: evaluation of concordance between genomic and transcriptional profiles. Eur J Cancer.

[28] Brannon AR, Vakiani E, Sylvester BE, Scott SN, McDermott G, Shah RH, Kania K, Viale A, Oschwald DM, Vacic V, Emde AK, Cercek A, Yaeger R (2014). Comparative sequencing analysis reveals high genomic concordance between matched primary and metastatic colorectal cancer lesions. Genome Biol.

[29] Bambury RM, Bhatt AS, Riester M, Pedamallu CS, Duke F, Bellmunt J, Stack EC, Werner L, Park R, Iyer G, Loda M, Kantoff PW, Michor F (2015). DNA copy number analysis of metastatic urothelial carcinoma with comparison to primary tumors. BMC Cancer.

[30] Vasan N, Yelensky R, Wang K, Moulder S, Dzimitrowicz H, Avritscher R, Wang B, Wu Y, Cronin MT, Palmer G, Symmans WF, Miller VA, Stephens P (2014). A targeted next-generation sequencing assay detects a high frequency of therapeutically targetable alterations in primary and metastatic breast cancers: implications for clinical practice. Oncologist.

[31] Adelaide J, Finetti P, Bekhouche I, Repellini L, Geneix J, Sircoulomb F, Charafe-Jauffret E, Cervera N, Desplans J, Parzy D, Schoenmakers E, Viens P, Jacquemier J (2007). Integrated profiling of basal and luminal breast cancers. Cancer Res.

[32] Olshen AB, Venkatraman ES, Lucito R, Wigler M (2004). Circular binary segmentation for the analysis of array-based DNA copy number data. Biostatistics.

[33] Mermel CH, Schumacher SE, Hill B, Meyerson ML, Beroukhim R, Getz G (2011). GISTIC2. 0 facilitates sensitive and confident localization of the targets of focal somatic copy-number alteration in human cancers. Genome Biol.

[34] Collette Y, Prebet T, Goubard A, Adelaide J, Castellano R, Carbuccia N, Garnier S, Guille A, Arnoulet C, Charbonier A, Mozziconacci MJ, Birnbaum D, Chaffanet M (2015). Drug response profiling can predict response to ponatinib in a patient with t(1;9)(q24;q34)-associated B-cell acute lymphoblastic leukemia. Blood Cancer J.

[35] Li H, Durbin R (2009). Fast and accurate short read alignment with Burrows-Wheeler transform. Bioinformatics.

[36] Garrison E, Marth G (2012). Haplotype-based variant detection from short-read sequencing.

[37] DePristo MA, Banks E, Poplin R, Garimella KV, Maguire JR, Hartl C, Philippakis AA, del Angel G, Rivas MA, Hanna M, McKenna A, Fennell TJ, Kernytsky AM (2011). A framework for variation discovery and genotyping using next-generation DNA sequencing data. Nat Genet.

[38] Robinson JT, Thorvaldsdottir H, Winckler W, Guttman M, Lander ES, Getz G, Mesirov JP (2011). Integrative genomics viewer. Nat Biotechnol.

[39] Thorvaldsdottir H, Robinson JT, Mesirov JP (2013). Integrative Genomics Viewer (IGV): high-performance genomics data visualization and exploration. Brief Bioinform.

[40] Suzuki R, Shimodaira H (2006). Pvclust: an R package for assessing the uncertainty in hierarchical clustering. Bioinformatics.

