# Supplementary material for: Comparative genomic analysis of primary tumors and metastases in breast cancer
Source: Oncotarget. 2016 Mar 25;7(19):27208–19. doi: 10.18632/oncotarget.8349 (PMC5053643; doi:10.18632/oncotarget.8349)
Supplement: Supplementary file 1 [file oncotarget-07-27208-s001.pdf]

Comparative genomic analysis of primary tumors and metastases in breast cancer

Supplementary Materials

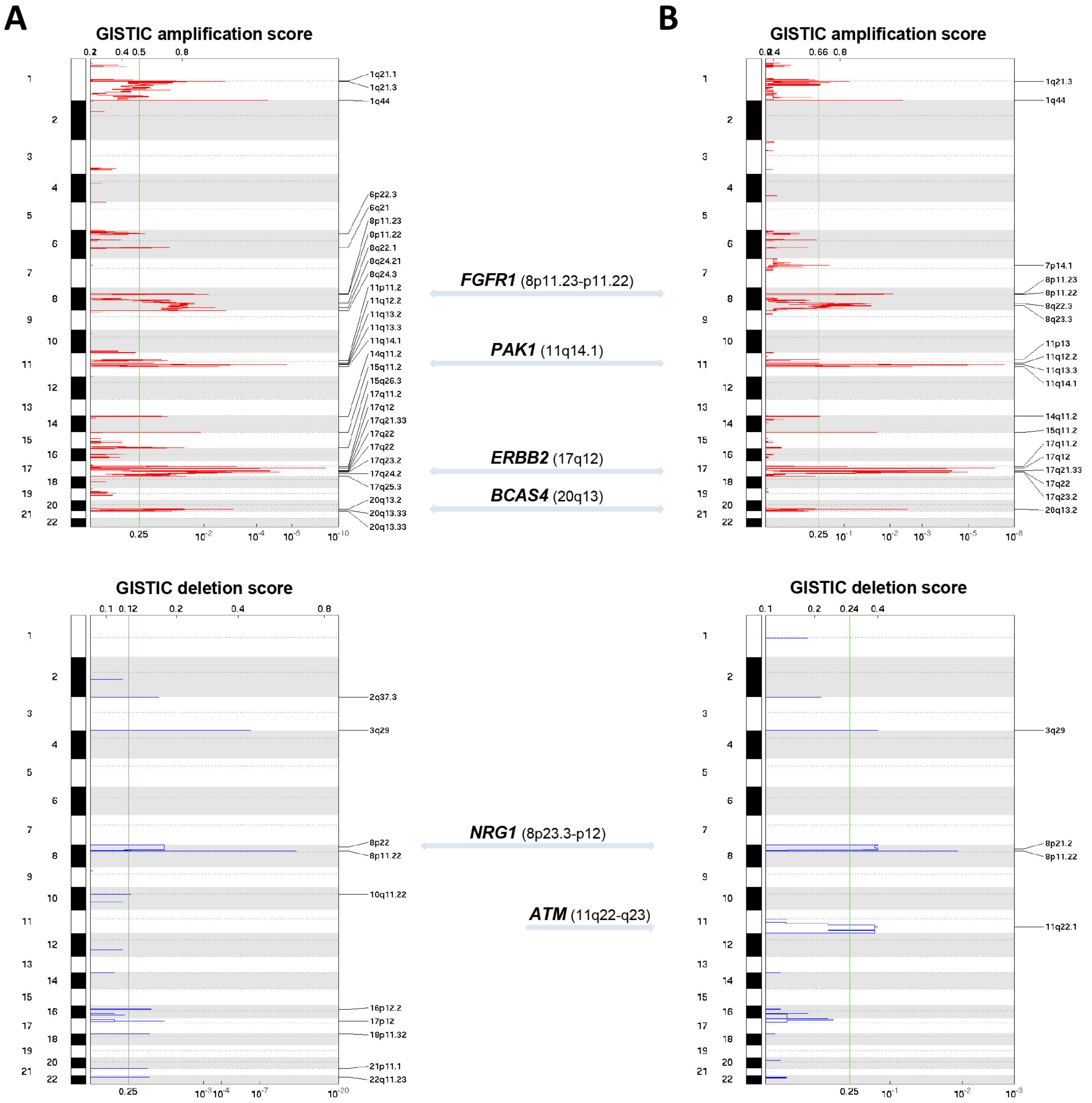

Supplementary Figure S1: GISTIC analysis of copy number profiles. The copy number profiles of primary tumors (**A**) and metastases (**B**) are shown. The x-axis represents the q-value (the vertical green line indicates the cut-off *q*-value of 0.25 for defining significant events) and the y-axis the chromosomal location. The GISTIC amplification (red peaks) or deletion (blue peaks) scores are shown at the top of each panel.

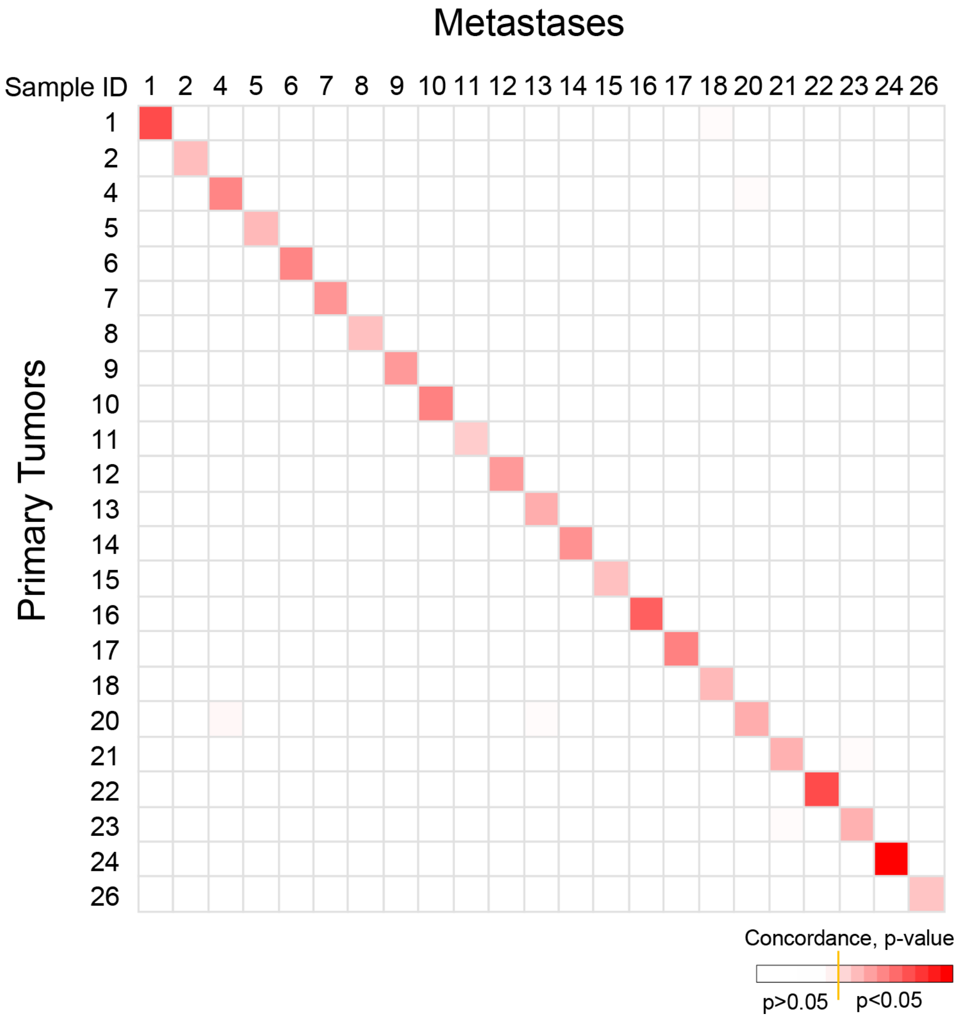

Supplementary Figure S2: Concordance analysis based on mutational profiles. Concordance matrix based on the $-\log_{10}$ *p*-values of the Fisher's exact test of a 2-by-2 contingency table representing the number of variants that are unshared or shared between each metastasis and each primary tumor. The test determines whether two samples share a significant number of variants. The *p*-value is color-coded according to the scale shown below the matrix.

Supplementary Table S1: Count of copy number alterations (CNA) in all samples

Sample ID	Type	Gain	Loss	Amplification	Deletion	Total CNA number	Total CNA Percent*
1	Primary Tumor	1539	14	588	0	2141	1.33%
	Metastasis	7579	279	1531	0	9389	5.83%
2	Primary Tumor	9176	91	519	0	9786	6.08%
	Metastasis	21537	20	9966	168	31691	19.68%
4	Primary Tumor	841	21	623	0	1485	0.92%
	Metastasis	7377	21	844	0	8242	5.12%
5	Primary Tumor	18262	546	33	0	18841	11.70%
	Metastasis	10816	19	8	0	10843	6.73%
6	Primary Tumor	6593	43	1514	12	8162	5.07%
	Metastasis	7309	148	1763	12	9232	5.73%
7	Primary Tumor	25029	81	7438	0	32548	20.21%
	Metastasis	68	0	22	0	90	0.06%
8	Primary Tumor	5116	0	112	0	5228	3.25%
	Metastasis	9252	0	832	0	10084	6.26%
9	Primary Tumor	7813	68	456	12	8349	5.18%
	Metastasis	20096	56	1610	12	21774	13.52%
10	Primary Tumor	11063	6	707	0	11776	7.31%
	Metastasis	841	711	11314	0	12866	7.99%
11	Primary Tumor	1381	0	294	0	1675	1.04%
	Metastasis	317	15	70	0	402	0.25%
12	Primary Tumor	8730	15	666	0	9411	5.84%
	Metastasis	16682	3396	964	7	21049	13.07%
13	Primary Tumor	18166	235	1919	12	20332	12.62%
	Metastasis	26948	6969	4029	191	38137	23.68%
14	Primary Tumor	22971	71	8067	0	31109	19.31%
	Metastasis	32745	27	2139	0	34911	21.67%
15	Primary Tumor	5531	24	729	6	6290	3.91%
	Metastasis	8839	14	931	0	9784	6.07%
16	Primary Tumor	6569	30	1029	0	7628	4.74%
	Metastasis	7436	127	1359	0	8922	5.54%
17	Primary Tumor	4298	20	14	11	4343	2.70%
	Metastasis	4218	11	8	12	4249	2.64%
18	Primary Tumor	7719	28	1072	0	8819	5.48%
	Metastasis	4446	20	734	0	5200	3.23%
20	Primary Tumor	12740	0	6763	7	19510	12.11%
	Metastasis	20938	7	3848	7	24800	15.40%
21	Primary Tumor	15506	72	325	0	15903	9.87%
	Metastasis	292	0	70	0	362	0.22%

22	Primary Tumor	4415	0	2540	0	6955	4.32%
	Metastasis	3947	0	2868	0	6815	4.23%
23	Primary Tumor	45558	6	8769	11	54344	33.74%
	Metastasis	366	0	8	0	374	0.23%
24	Primary Tumor	7671	24	57	6	7758	4.82%
	Metastasis	11952	2206	65	6	14229	8.83%
26	Primary Tumor	5994	0	348	0	6342	3.94%
	Metastasis	94	0	113	0	207	0.13%

*the percentage of probes displaying a CNA per sample was calculated as the total number of probes with CNAs divided by the total number of probes.

Supplementary Table S2: Details of all detected mutations in all samples

Sample ID	Type	Gene	Variant Allele Frequency	Mutation_cDNA	Mutation_Protein	Class	Recurrent
1	Primary Tumor	ARID1A	54%	c.C1258T	p.Q420X	stopgain SNV	no
1	Primary Tumor	USH2A	30%	c.C12361T	p.R4121C	nonsynonymous SNV	no
1	Primary Tumor	WT1	22%	c.G326C	p.G109A	nonsynonymous SNV	no
1	Primary Tumor	MLL	19%	c.G6745A	p.V2249I	nonsynonymous SNV	no
1	Primary Tumor	ERBB3	52%	c.T3286C	p.S1096P	nonsynonymous SNV	no
1	Primary Tumor	PALB2	25%	c.C2590T	p.P864S	nonsynonymous SNV	no
1	Primary Tumor	ERCC3	11%	c.A248G	p.H83R	nonsynonymous SNV	no
1	Primary Tumor	PIK3CA	49%	c.A3140T	p.H1047L	nonsynonymous SNV	yes
1	Primary Tumor	MLL3	15%	c.C2689T	p.R897X	stopgain SNV	no
1	Primary Tumor	AFF2	57%	c.G1858C	p.A620P	nonsynonymous SNV	no
1	Primary Tumor	BARD1	49%	c.1075_1095del	p.359_365del	nonframeshift deletion	no
1	Metastasis	ARID1A	49%	c.C1258T	p.Q420X	stopgain SNV	no
1	Metastasis	USH2A	75%	c.C12361T	p.R4121C	nonsynonymous SNV	no
1	Metastasis	WT1	21%	c.G326C	p.G109A	nonsynonymous SNV	no
1	Metastasis	MLL	32%	c.G6745A	p.V2249I	nonsynonymous SNV	no
1	Metastasis	ERBB3	60%	c.T3286C	p.S1096P	nonsynonymous SNV	no
1	Metastasis	PALB2	66%	c.C2590T	p.P864S	nonsynonymous SNV	no
1	Metastasis	ERCC3	25%	c.A248G	p.H83R	nonsynonymous SNV	no
1	Metastasis	PIK3CA	48%	c.A3140T	p.H1047L	nonsynonymous SNV	yes
1	Metastasis	MLL3	43%	c.C2689T	p.R897X	stopgain SNV	no
1	Metastasis	AFF2	60%	c.G1858C	p.A620P	nonsynonymous SNV	no
1	Metastasis	BARD1	52%	c.1075_1095del	p.359_365del	nonframeshift deletion	no
2	Primary Tumor	USH2A	3%	c.A15496G	p.I5166V	nonsynonymous SNV	no
2		RB1(NM_000321	43%			splicing	no
2		TP53	17%	c.G422T	p.R141L	nonsynonymous SNV	yes
2		APOB	47%	c.C4755A	p.N1585K	nonsynonymous SNV	no

2		XPC	12%	c.G661T	p.V221L	nonsynonymous SNV	no
2		TBC1D1	24%	c.T311A	p.L104Q	nonsynonymous SNV	no
2		KIT	49%	c.T2771C	p.I924T	nonsynonymous SNV	no
2		TAP1	33%	c.185_187del	p.62_63del	nonframeshift deletion	no
2	Metastasis	USH2A	24%	c.A15496G	p.I5166V	nonsynonymous SNV	no
2		TP53	23%	c.G422T	p.R141L	nonsynonymous SNV	yes
2		ITGA4	54%	c.T65C	p.L22P	nonsynonymous SNV	no
2		XPC	10%	c.G661T	p.V221L	nonsynonymous SNV	no
2		TBC1D1	24%	c.T311A	p.L104Q	nonsynonymous SNV	no
2		KIT	34%	c.T2771C	p.I924T	nonsynonymous SNV	no
2		TRIM23	25%	c.C668G	p.A223G	nonsynonymous SNV	no
2		TRIM23	63%	c.T648A	p.H216Q	nonsynonymous SNV	no
2		CCND3	27%	c.C562G	p.Q188E	nonsynonymous SNV	no
2		POLE	20%	c.4780_4788del	p.1594_1596del	nonframeshift deletion	no
2		NOTCH4	80%	c.2811_2844del	p.937_948del	frameshift deletion	no
2		TAP1	11%	c.185_187del	p.62_63del	nonframeshift deletion	no
4	Primary Tumor	NOTCH2	31%	c.A7304G	p.D2435G	nonsynonymous SNV	no
4		PARP1	23%	c.G249T	p.Q83H	nonsynonymous SNV	no
4		PRF1	48%	c.A755G	p.N252S	nonsynonymous SNV	no
4		FANCM	50%	c.G2257T	p.D753Y	nonsynonymous SNV	no
4		TP53	35%	c.T304C	p.Y102H	nonsynonymous SNV	yes
4		JAK3	49%	c.C62T	p.T21M	nonsynonymous SNV	no
4		PIK3CA	7%	c.A3140G	p.H1047R	nonsynonymous SNV	yes
4		RECQL4	31%	c.G2252A	p.R751Q	nonsynonymous SNV	no
4		FANCB	48%	c.G554T	p.G185V	nonsynonymous SNV	no
4		RB1	11%	c.211_214del	p.71_72del	frameshift deletion	no

4		CEBPA	71%	c.589_590insACCCGC	p.P197delinsHPP	nonframeshift insertion	no
4		MAP3K4	24%	c.81_82insCCG	p.P27delinsPP	nonframeshift insertion	no
4	Metastasis	NOTCH2	76%	c.A7304G	p.D2435G	nonsynonymous SNV	no
4		PRF1	23%	c.A755G	p.N252S	nonsynonymous SNV	no
4		MLL(NM_005933	46%			splicing	no
4		FANCM	55%	c.G2257T	p.D753Y	nonsynonymous SNV	no
4		TP53	46%	c.T304C	p.Y102H	nonsynonymous SNV	yes
4		MUC16	33%	c.G17356A	p.D5786N	nonsynonymous SNV	no
4		JAK3	32%	c.C62T	p.T21M	nonsynonymous SNV	no
4		SMARCB1	50%	c.G591A	p.W197X	stopgain SNV	no
4		PIK3CA	47%	c.A3140G	p.H1047R	nonsynonymous SNV	yes
4		RECQL4	74%	c.G2252A	p.R751Q	nonsynonymous SNV	no
4		FANCB	46%	c.G554T	p.G185V	nonsynonymous SNV	no
4		NF1	86%	c.2027dupC	p.T676fs	frameshift insertion	no
4		CEBPA	38%	c.589_590insACCCGC	p.P197delinsHPP	nonframeshift insertion	no
4		MAP3K4	63%	c.81_82insCCG	p.P27delinsPP	nonframeshift insertion	no
5	Primary Tumor	MTOR	36%	c.A2134G	p.I712V	nonsynonymous SNV	no
5		IL6R	12%	c.T829C	p.C277R	nonsynonymous SNV	no
5		USH2A	9%	c.A8245G	p.I2749V	nonsynonymous SNV	no
5		PARP1	3%	c.C193T	p.R65W	nonsynonymous SNV	no
5		PTPN11	31%	c.T854C	p.F285S	nonsynonymous SNV	no
5		FANCI	7%	c.T3357G	p.Y1119X	stopgain SNV	no
5		CREBBP	39%	c.A4546G	p.K1516E	nonsynonymous SNV	no
5		PLK1	8%	c.G1648A	p.V550M	nonsynonymous SNV	no
5		CTCF	54%	c.C997T	p.Q333X	stopgain SNV	no
5		NF1	11%	c.G5021A	p.R1674Q	nonsynonymous SNV	no
5		SMARCA4	3%	c.G2654A	p.R885H	nonsynonymous SNV	no
5		ARHGAP35	5%	c.C362T	p.A121V	nonsynonymous SNV	no

5		ARHGAP35	55%	c.G457A	p.G153R	nonsynonymous SNV	no
5		MYCN	52%	c.G212A	p.S71N	nonsynonymous SNV	no
5		CHEK2	18%	c.C283T	p.R95X	stopgain SNV	no
5		PIK3CA	21%	c.A3140G	p.H1047R	nonsynonymous SNV	yes
5		NSD1	45%	c.C5956T	p.R1986C	nonsynonymous SNV	no
5		CAP2	9%	c.C1225T	p.P409S	nonsynonymous SNV	no
5		ARID1B	6%	c.G2611T	p.G871X	stopgain SNV	no
5		ARID1B	69%	c.C6415T	p.Q2139X	stopgain SNV	no
5		MET	15%	c.C2975T	p.T992I	nonsynonymous SNV	no
5		MLL3	32%	c.G12569A	p.S4190N	nonsynonymous SNV	no
5		EXT1	60%	c.A2219G	p.Y740C	nonsynonymous SNV	no
5		SELP	5%	c.984_985insAA	p.A329fs	frameshift insertion	no
5		BLM	5%	c.1536delA	p.G512fs	frameshift deletion	no
5		MSH6	68%	c.3254dupC	p.T1085fs	frameshift insertion	no
5		MAP3K1	16%	c.2668delC	p.P890fs	frameshift deletion	no
5		TEK	17%	c.2980delA	p.K994fs	frameshift deletion	no
5		NOTCH1	3%	c.4732_4734del	p.1578_1578del	nonframeshift deletion	no
5	Metastasis	MTOR	13%	c.A2134G	p.I712V	nonsynonymous SNV	no
5		PARP1	21%	c.C193T	p.R65W	nonsynonymous SNV	no
5		MLL2	39%	c.G14243A	p.S4748N	nonsynonymous SNV	no
5		CTCF	30%	c.C997T	p.Q333X	stopgain SNV	no
5		TP53	52%	c.C421T	p.R141C	nonsynonymous SNV	yes
5		SMAD4	14%	c.C821T	p.A274V	nonsynonymous SNV	no
5		ARHGAP35	11%	c.C362T	p.A121V	nonsynonymous SNV	no
5		DNER	24%	c.T1807C	p.Y603H	nonsynonymous SNV	no
5		CHEK2	51%	c.C283T	p.R95X	stopgain SNV	no
5		PIK3CA	28%	c.A3140G	p.H1047R	nonsynonymous SNV	yes
5		MET	29%	c.C2975T	p.T992I	nonsynonymous SNV	no

5		MLL3	40%	c.A13442G	p.N4481S	nonsynonymous SNV	no
5		MLL3	20%	c.G12569A	p.S4190N	nonsynonymous SNV	no
5		ANGPT2	16%	c.T1177C	p.W393R	nonsynonymous SNV	no
5		TNFRSF10A	44%	c.A352G	p.I118V	nonsynonymous SNV	no
5		SELP	15%	c.984_985insAA	p.A329fs	frameshift insertion	no
5		FLT3	48%	c.1863dupT	p.G622fs	frameshift insertion	no
5		BLM	32%	c.1536delA	p.G512fs	frameshift deletion	no
5		ZFHX3	55%	c.1037dupA	p.N346fs	frameshift insertion	no
5		MSH6	67%	c.3253_3254insCC	p.T1085fs	frameshift insertion	no
5		MAP3K1	18%	c.2668delC	p.P890fs	frameshift deletion	no
5		FGFR4	16%	c.1578delC	p.R526fs	frameshift deletion	no
5		NBN	61%	c.1396delA	p.R466fs	frameshift deletion	no
5		NOTCH1	23%	c.4732_4734del	p.1578_1578del	nonframeshift deletion	no
6	Primary Tumor	SELP	19%	c.C1229A	p.T410N	nonsynonymous SNV	no
6		PARP1	20%	c.G2413A	p.D805N	nonsynonymous SNV	no
6		NCOR2	46%	c.A2365G	p.T789A	nonsynonymous SNV	no
6		CDH1	28%	c.C187T	p.R63X	stopgain SNV	no
6		NF1	69%	c.G847T	p.D283Y	nonsynonymous SNV	no
6		MUC16	34%	c.G40547A	p.R13516K	nonsynonymous SNV	no
6		MUC16	51%	c.C22046T	p.P7349L	nonsynonymous SNV	no
6		PDGFRB	4%	c.G1487A	p.R496H	nonsynonymous SNV	no
6		EGFL7	41%	c.G379A	p.A127T	nonsynonymous SNV	no
6		CDH1	58%	c.110dupA	p.Y37_T38delinsX	stopgain SNV	no
6		ZFHX3	21%	c.6886_6887insAGCAACCACAGGTGCAGCAGC	p.P2296delinsQQPQVQQP	nonframeshift insertion	no
6	Metastasis	SELP	36%	c.C1229A	p.T410N	nonsynonymous SNV	no
6		USH2A	47%	c.C8263G	p.L2755V	nonsynonymous SNV	no
6		PARP1	38%	c.G2413A	p.D805N	nonsynonymous SNV	no

6		NCOR2	49%	c.A2365G	p.T789A	nonsynonymous SNV	no
6		POLE	22%	c.G6784T	p.A2262S	nonsynonymous SNV	no
6		NF1	77%	c.G847T	p.D283Y	nonsynonymous SNV	no
6		SMAD4	33%	c.C725G	p.S242X	stopgain SNV	no
6		MUC16	55%	c.G40547A	p.R13516K	nonsynonymous SNV	no
6		MUC16	9%	c.C22046T	p.P7349L	nonsynonymous SNV	no
6		DIAPH1	40%	c.G2948A	p.R983K	nonsynonymous SNV	no
6		PDGFRB	33%	c.G1487A	p.R496H	nonsynonymous SNV	no
6		EGFL7	64%	c.G379A	p.A127T	nonsynonymous SNV	no
6		ATRX	52%	c.T965A	p.I322N	nonsynonymous SNV	no
6		CDH1	18%	c.110dupA	p.Y37_T38delinsX	stopgain SNV	no
6		ZFHX3	16%	c.6886_6887insAGCAACCACAGGTGCAGCAGC	p.P2296delinsQQPQVQQP	nonframeshift insertion	no
7	Primary Tumor	CD248	44%	c.C191T	p.P64L	nonsynonymous SNV	no
7		BRCA2	78%	c.G4585A	p.G1529R	nonsynonymous SNV	no
7		BRCA2	22%	c.A9976T	p.K3326X	stopgain SNV	no
7		TP53	39%	c.G128A	p.R43H	nonsynonymous SNV	yes
7		MUC16	21%	c.C16572G	p.I5524M	nonsynonymous SNV	no
7		SMARCA4	49%	c.C2559A	p.F853L	nonsynonymous SNV	no
7		XPC	48%	c.C146G	p.S49C	nonsynonymous SNV	no
7		MLLT4	46%	c.G4600T	p.A1534S	nonsynonymous SNV	no
7		EPHA2	22%	c.987delC	p.P329fs	frameshift deletion	no
7		RB1	47%	c.1193delA	p.E398fs	frameshift deletion	no
7		ARID1B	45%	c.339_340insCAG	p.F113delinsFQ	nonframeshift insertion	no
7	Metastasis	BRCA2	45%	c.G4585A	p.G1529R	nonsynonymous SNV	no
7		BRCA2	44%	c.A9976T	p.K3326X	stopgain SNV	no
7		TP53	46%	c.G128A	p.R43H	nonsynonymous SNV	yes
7		MUC16	48%	c.C16572G	p.I5524M	nonsynonymous SNV	no

7		SMARCA4	53%	c.C2559A	p.F853L	nonsynonymous SNV	no
7		MYH9	3%	c.G1425C	p.K475N	nonsynonymous SNV	no
7		XPC	3%	c.C146G	p.S49C	nonsynonymous SNV	no
7		MLLT4	2%	c.G4600T	p.A1534S	nonsynonymous SNV	no
7		ARID1B	4%	c.339_340insCAG	p.F113delinsFQ	nonframeshift insertion	no
8	Primary Tumor	MTOR	6%	c.C1393T	p.P465S	nonsynonymous SNV	no
8		FOXA1	46%	c.A586G	p.I196V	nonsynonymous SNV	no
8		SMARCA4	17%	c.C622T	p.R208W	nonsynonymous SNV	no
8		CD22	20%	c.G896T	p.G299V	nonsynonymous SNV	no
8		SOS1	48%	c.C3257G	p.P1086R	nonsynonymous SNV	no
8		PIK3CA	36%	c.G1633A	p.E545K	nonsynonymous SNV	yes
8		PMS2	3%	c.C1437G	p.H479Q	nonsynonymous SNV	no
8		CUX1	54%	c.C286T	p.L96F	nonsynonymous SNV	no
8		MLL3	15%	c.G6695A	p.G2232D	nonsynonymous SNV	no
8		PTCH1	17%	c.C3317T	p.T1106M	nonsynonymous SNV	no
8		MEN1	42%	c.312_313del	p.104_105del	frameshift deletion	no
8		SOX17	23%	c.948_949insCACCAG	p.Q316delinsQHQ	nonframeshift insertion	no
8	Metastasis	MTOR	43%	c.C1393T	p.P465S	nonsynonymous SNV	no
8		SELP	52%	c.C393G	p.C131W	nonsynonymous SNV	no
8		MEN1	40%	c.A1477T	p.K493X	stopgain SNV	no
8		SOS1	5%	c.C3257G	p.P1086R	nonsynonymous SNV	no
8		PMS2	37%	c.C1437G	p.H479Q	nonsynonymous SNV	no
8		CUX1	49%	c.C286T	p.L96F	nonsynonymous SNV	no
8		MLL3	7%	c.G6695A	p.G2232D	nonsynonymous SNV	no
8		MLL3	40%	c.C3114A	p.D1038E	nonsynonymous SNV	no
8		SOX17	12%	c.948_949insCACCAG	p.Q316delinsQHQ	nonframeshift insertion	no
9	Primary Tumor	USH2A	53%	c.A6347G	p.H2116R	nonsynonymous SNV	no

9		BRCA2	57%	c.A6347G	p.H2116R	nonsynonymous SNV	no
9		NOTCH3	26%	c.G4755T	p.E1585D	nonsynonymous SNV	no
9		APOB	47%	c.A4181G	p.D1394G	nonsynonymous SNV	no
9		PIK3CA	41%	c.G353A	p.G118D	nonsynonymous SNV	yes
9		NSD1	23%	c.G3056A	p.R1019H	nonsynonymous SNV	no
9		NBN	38%	c.C643T	p.R215W	nonsynonymous SNV	no
9		FANCG	17%	c.G934A	p.V312I	nonsynonymous SNV	no
9		CDKN2A	56%	c.9_32del	p.3_11del	nonframeshift deletion	no
9	Metastasis	ARID1A	46%	c.G232T	p.E78X	stopgain SNV	no
9		ARID1A	25%	c.G5326A	p.E1776K	nonsynonymous SNV	no
9		ARID1A	18%	c.G5335T	p.E1779X	stopgain SNV	no
9		USH2A	18%	c.A6347G	p.H2116R	nonsynonymous SNV	no
9		SIRT1	11%	c.C704A	p.T235K	nonsynonymous SNV	no
9		BRCA2	75%	c.A6347G	p.H2116R	nonsynonymous SNV	no
9		NOTCH3	48%	c.G4755T	p.E1585D	nonsynonymous SNV	no
9		APOB	40%	c.A4181G	p.D1394G	nonsynonymous SNV	no
9		MSH2	30%	c.G900A	p.M300I	nonsynonymous SNV	no
9		BAP1	52%	c.G1492C	p.E498Q	nonsynonymous SNV	no
9		PIK3CA	11%	c.G353A	p.G118D	nonsynonymous SNV	yes
9		PIK3CA	29%	c.G2176A	p.E726K	nonsynonymous SNV	no
9		NSD1	39%	c.G3056A	p.R1019H	nonsynonymous SNV	no
9		NOTCH4	31%	c.G1531A	p.E511K	nonsynonymous SNV	no
9		VEGFA	18%	c.G403T	p.A135S	nonsynonymous SNV	no
9		ESR1	28%	c.G1086C	p.K362N	nonsynonymous SNV	no
9		NBN	29%	c.C643T	p.R215W	nonsynonymous SNV	no
9		FANCG	14%	c.G934A	p.V312I	nonsynonymous SNV	no
9		CDH1	56%	c.80_81insCT	p.P27fs	frameshift insertion	no

9		CDKN2A	25%	c.9_32del	p.3_11del	nonframeshift deletion	no
10	Primary Tumor	SDHB	38%	c.T487C	p.S163P	nonsynonymous SNV	no
10		AKT1	37%	c.G130A	p.D44N	nonsynonymous SNV	no
10		AKT1	51%	c.G49A	p.E17K	nonsynonymous SNV	yes
10		ITGAL	16%	c.A103G	p.S35G	nonsynonymous SNV	no
10		JAK3	39%	c.A757G	p.T253A	nonsynonymous SNV	no
10		CD22	53%	c.A1028C	p.H343P	nonsynonymous SNV	no
10		TPX2	26%	c.C20A	p.S7Y	nonsynonymous SNV	no
10		EP300	52%	c.G3787T	p.E1263X	stopgain SNV	no
10		FAT1	47%	c.A5320G	p.I1774V	nonsynonymous SNV	no
10	Metastasis	SDHB	95%	c.T487C	p.S163P	nonsynonymous SNV	no
10		AKT1	95%	c.G130A	p.D44N	nonsynonymous SNV	no
10		AKT1	68%	c.G49A	p.E17K	nonsynonymous SNV	yes
10		ITGAL	31%	c.A103G	p.S35G	nonsynonymous SNV	no
10		JAK3	31%	c.A757G	p.T253A	nonsynonymous SNV	no
10		CD22	13%	c.A1028C	p.H343P	nonsynonymous SNV	no
10		TPX2	46%	c.C20A	p.S7Y	nonsynonymous SNV	no
10		FAT1	32%	c.A5320G	p.I1774V	nonsynonymous SNV	no
11	Primary Tumor	ANK3	57%	c.T2544A	p.D848E	nonsynonymous SNV	no
11		TP53	49%	c.G317A	p.C106Y	nonsynonymous SNV	yes
11		MUC16	48%	c.G6967T	p.A2323S	nonsynonymous SNV	no
11		MLL3	13%	c.C11381G	p.P3794R	nonsynonymous SNV	no
11		PTEN	18%	c.281_287del	p.94_96del	frameshift deletion	no
11		NCAM1	19%	c.481dupA	p.L160fs	frameshift insertion	no
11	Metastasis	ANK3	52%	c.T2544A	p.D848E	nonsynonymous SNV	no
11		SACS	ND	c.G8900A	p.R2967H	nonsynonymous SNV	no
11		TP53	47%	c.G317A	p.C106Y	nonsynonymous SNV	yes

11		MUC16	51%	c.G6967T	p.A2323S	nonsynonymous SNV	no
11		MLL3	13%	c.C11381G	p.P3794R	nonsynonymous SNV	no
11		GNAS	6%	c.1189_1190insTGACGCCCCAGCCGATCCCGACTCCGGGGCGGCCCG	p.L397delinsLTPQPIPTPGRPV	nonframeshift insertion	no
12	Primary Tumor	USH2A	12%	c.T7685C	p.V2562A	nonsynonymous SNV	no
12		SACS	62%	c.T11739G	p.D3913E	nonsynonymous SNV	no
12		MSH6	44%	c.G431T	p.S144I	nonsynonymous SNV	no
12		ITGAV	25%	c.C1510T	p.R504X	stopgain SNV	no
12		PIK3CA	24%	c.G1624A	p.E542K	nonsynonymous SNV	yes
12		GATA3	34%	c.994_995insC	p.N332fs	frameshift insertion	no
12	Metastasis	USH2A	17%	c.T7685C	p.V2562A	nonsynonymous SNV	no
12		SACS	50%	c.T11739G	p.D3913E	nonsynonymous SNV	no
12		MSH6	39%	c.G431T	p.S144I	nonsynonymous SNV	no
12		ITGAV	67%	c.C1510T	p.R504X	stopgain SNV	no
12		PIK3CA	19%	c.G1624A	p.E542K	nonsynonymous SNV	yes
12		GATA3	41%	c.994_995insC	p.N332fs	frameshift insertion	no
13	Primary Tumor	GATA3	31%	c.T100G	p.S34A	nonsynonymous SNV	no
13		TP53	23%	c.T259G	p.Y87D	nonsynonymous SNV	no
13		MUC16	13%	c.A19619G	p.H6540R	nonsynonymous SNV	no
13		DNER	73%	c.G2011C	p.G671R	nonsynonymous SNV	no
13		PIK3CA	41%	c.A3140G	p.H1047R	nonsynonymous SNV	yes
13		PTCH1	15%	c.G3890A	p.R1297Q	nonsynonymous SNV	no
13		KDM6A	61%	c.T3599G	p.L1200R	nonsynonymous SNV	no
13		TBX3	53%	c.951_952insA	p.E318fs	frameshift insertion	no
13	Metastasis	GATA3	20%	c.T100G	p.S34A	nonsynonymous SNV	no
13		TP53	44%	c.T259G	p.Y87D	nonsynonymous SNV	no
13		MUC16	91%	c.A19619G	p.H6540R	nonsynonymous SNV	no
13		NOTCH3	12%	c.G4285A	p.E1429K	nonsynonymous SNV	no

13		DNER	46%	c.G2011C	p.G671R	nonsynonymous SNV	no
13		PIK3CA	81%	c.A3140G	p.H1047R	nonsynonymous SNV	yes
13		TBX3	75%	c.951_952insA	p.E318fs	frameshift insertion	no
14	Primary Tumor	EXT2	18%	c.G683T	p.G228V	nonsynonymous SNV	no
14		POLE	60%	c.T4418C	p.L1473P	nonsynonymous SNV	no
14		SOS1	19%	c.G2748T	p.L916F	nonsynonymous SNV	no
14		NOTCH4	37%	c.G5693C	p.R1898P	nonsynonymous SNV	no
14		ROS1	31%	c.A2672T	p.Y891F	nonsynonymous SNV	no
14		JAK2	18%	c.A967G	p.I323V	nonsynonymous SNV	no
14		TP53	71%	c.438_445del	p.146_149del	frameshift deletion	no
14		TP53	70%	c.433_436del	p.145_146del	frameshift deletion	no
14	Metastasis	EXT2	21%	c.G683T	p.G228V	nonsynonymous SNV	no
14		POLE	48%	c.T4418C	p.L1473P	nonsynonymous SNV	no
14		NOTCH4	18%	c.G5693C	p.R1898P	nonsynonymous SNV	no
14		ROS1	30%	c.A2672T	p.Y891F	nonsynonymous SNV	no
14		JAK2	26%	c.A967G	p.I323V	nonsynonymous SNV	no
14		TP53	45%	c.438_445del	p.146_149del	frameshift deletion	no
14		TP53	45%	c.433_436del	p.145_146del	frameshift deletion	no
15	Primary Tumor	FLT4	33%	c.C2860T	p.P954S	nonsynonymous SNV	no
15		PTCH1	53%	c.G192C	p.Q64H	nonsynonymous SNV	no
15		GATA3	6%	c.989dupG	p.R330fs	frameshift insertion	no
15		CEBPA	49%	c.589_590insACCCGC	p.P197delinsHPP	nonframeshift insertion	no
15	Metastasis	PTEN	46%	c.C733T	p.Q245X	stopgain SNV	no
15		FLT4	55%	c.C2860T	p.P954S	nonsynonymous SNV	no
15		PTCH1	8%	c.G192C	p.Q64H	nonsynonymous SNV	no
15		GATA3	46%	c.989dupG	p.R330fs	frameshift insertion	no
15		CEBPA	30%	c.589_590insACCCGC	p.P197delinsHPP	nonframeshift insertion	no

16	Primary Tumor	NTRK1	40%	c.G1564A	p.E522K	nonsynonymous SNV	no
16		ANK3	10%	c.G10408A	p.E3470K	nonsynonymous SNV	no
16		ATM	75%	c.C146G	p.S49C	nonsynonymous SNV	no
16		MLL	7%	c.C6797T	p.S2266F	nonsynonymous SNV	no
16		CDKN1B	13%	c.C413G	p.S138W	nonsynonymous SNV	no
16		MLL2	8%	c.C12101T	p.S4034F	nonsynonymous SNV	no
16		SACS	58%	c.C10999T	p.H3667Y	nonsynonymous SNV	no
16		DICER1	19%	c.G4360C	p.D1454H	nonsynonymous SNV	no
16		FANCI	5%	c.T3454G	p.F1152V	nonsynonymous SNV	no
16		TSC2	9%	c.G700T	p.E234X	stopgain SNV	no
16		ITGAL	12%	c.G1525A	p.E509K	nonsynonymous SNV	no
16		TP53	61%	c.C61T	p.P21S	nonsynonymous SNV	no
16		GNAS	5%	c.C421T	p.P141S	nonsynonymous SNV	no
16		MYH9	8%	c.C5408T	p.S1803F	nonsynonymous SNV	no
16		MYH9	34%	c.G2785A	p.E929K	nonsynonymous SNV	no
16		EP300	8%	c.G4653C	p.K1551N	nonsynonymous SNV	no
16		PIK3CA	32%	c.C1616G	p.P539R	nonsynonymous SNV	yes
16		MAP3K4	40%	c.C3801G	p.I1267M	nonsynonymous SNV	no
16		RECQL4	11%	c.G3331C	p.E1111Q	nonsynonymous SNV	no
16		PTPRD	8%	c.A1932C	p.E644D	nonsynonymous SNV	no
16		AMER1	6%	c.A3388G	p.N1130D	nonsynonymous SNV	no
16		FAT1	30%	c.3038_3053del	p.1013_1018del	frameshift deletion	no
16		SOX17	14%	c.948_949insCACCAG	p.Q316delinsQHQ	nonframeshift insertion	no
16	Metastasis	JAK1	42%	c.A2579C	p.K860T	nonsynonymous SNV	no
16		NTRK1	17%	c.G1564A	p.E522K	nonsynonymous SNV	no
16		ANK3	68%	c.G10408A	p.E3470K	nonsynonymous SNV	no
16		HRAS	16%	c.C461G	p.S154C	nonsynonymous SNV	no

16		WT1	15%	c.C59T	p.S20F	nonsynonymous SNV	no
16		ATM	16%	c.C146G	p.S49C	nonsynonymous SNV	no
16		MLL	52%	c.C6797T	p.S2266F	nonsynonymous SNV	no
16		MLL2	34%	c.C12101T	p.S4034F	nonsynonymous SNV	no
16		DICER1	23%	c.G4360C	p.D1454H	nonsynonymous SNV	no
16		FANCI	13%	c.T3454G	p.F1152V	nonsynonymous SNV	no
16		TSC2	65%	c.G700T	p.E234X	stopgain SNV	no
16		ERCC4	15%	c.G2284C	p.D762H	nonsynonymous SNV	no
16		TP53	31%	c.C61T	p.P21S	nonsynonymous SNV	no
16		MYH9	9%	c.G2785A	p.E929K	nonsynonymous SNV	no
16		EP300	46%	c.C724G	p.L242V	nonsynonymous SNV	no
16		PIK3CA	54%	c.C1616G	p.P539R	nonsynonymous SNV	yes
16		RECQL4	17%	c.G3331C	p.E1111Q	nonsynonymous SNV	no
16		PTPRD	5%	c.A1932C	p.E644D	nonsynonymous SNV	no
16		AMER1	30%	c.A3388G	p.N1130D	nonsynonymous SNV	no
16		FAT1	17%	c.3038_3053del	p.1013_1018del	frameshift deletion	no
16		SOX17	12%	c.948_949insCACCAG	p.Q316delinsQHQ	nonframeshift insertion	no
17	Primary Tumor	ANK3	21%	c.G2202T	p.Q734H	nonsynonymous SNV	no
17		SDHD	20%	c.G34A	p.V12I	nonsynonymous SNV	no
17		CHD4(NM_001273	44%			splicing	no
17		MLL2	20%	c.G8489A	p.R2830Q	nonsynonymous SNV	no
17		AKT1	11%	c.G49A	p.E17K	nonsynonymous SNV	yes
17		FES	7%	c.C1771T	p.R591C	nonsynonymous SNV	no
17		CDH1	53%	c.C67T	p.Q23X	stopgain SNV	no
17		NF1(NM_000267	28%			splicing	no
17		ROS1	18%	c.C2232G	p.S744R	nonsynonymous SNV	no
17		RECQL4	18%	c.C2920G	p.L974V	nonsynonymous SNV	no
17		BCOR	44%	c.C247T	p.R83W	nonsynonymous SNV	no

17		CDKN1B	41%	c.280dupC	p.R93fs	frameshift insertion	no
17		MLL3	47%	c.13896_13897insAGGT	p.V4633fs	frameshift insertion	no
17	Metastasis	SIRT1	23%	c.C202A	p.H68N	nonsynonymous SNV	no
17		SDHD	ND	c.G34A	p.V12I	nonsynonymous SNV	no
17		CHD4(NM_001273	46%			splicing	no
17		MLL2	18%	c.G8489A	p.R2830Q	nonsynonymous SNV	no
17		AKT1	10%	c.G49A	p.E17K	nonsynonymous SNV	yes
17		FES	49%	c.C1771T	p.R591C	nonsynonymous SNV	no
17		CDH1	29%	c.C67T	p.Q23X	stopgain SNV	no
17		ROS1	22%	c.C2232G	p.S744R	nonsynonymous SNV	no
17		RECQL4	42%	c.C2920G	p.L974V	nonsynonymous SNV	no
17		BCOR	51%	c.C247T	p.R83W	nonsynonymous SNV	no
17		ARID2	46%	c.405_406insCACAGTG TGTCGCTGGTGCGGT CTCATCTTCCTACAAT TACCAGCAT	p.Q135fs	frameshift insertion	no
17		MLL3	20%	c.13896_13897insAGGT	p.V4633fs	frameshift insertion	no
18	Primary Tumor	AXL	47%	c.G1412A	p.R471H	nonsynonymous SNV	no
18		CTNNB1	45%	c.T185C	p.V62A	nonsynonymous SNV	no
18		BAP1	17%	c.G1643A	p.R548H	nonsynonymous SNV	no
18		PIK3CA	14%	c.A3140T	p.H1047L	nonsynonymous SNV	yes
18		APC	45%	c.G3895C	p.E1299Q	nonsynonymous SNV	no
18		NOTCH4	42%	c.A1360C	p.N454H	nonsynonymous SNV	no
18		BRAF	11%	c.G772T	p.G258C	nonsynonymous SNV	no
18		VEGFB	24%	c.468_473del	p.156_158del	nonframeshift deletion	no
18		TP53	43%	c.261dupC	p.Y88fs	frameshift insertion	no
18	Metastasis	CTNNB1	43%	c.T185C	p.V62A	nonsynonymous SNV	no
18		PIK3CA	43%	c.A3140T	p.H1047L	nonsynonymous SNV	yes
18		APC	12%	c.G3895C	p.E1299Q	nonsynonymous SNV	no

18		VEGFB	14%	c.468_473del	p.156_158del	nonframeshift deletion	no
18		TP53	51%	c.261dupC	p.Y88fs	frameshift insertion	no
20	Primary Tumor	SACS	58%	c.G12346T	p.D4116Y	nonsynonymous SNV	no
20		MAX	22%	c.296-1G>T		splicing	no
20		ERCC4	53%	c.T2551G	p.F851V	nonsynonymous SNV	no
20		ARHGAP35	40%	c.T3284G	p.V1095G	nonsynonymous SNV	no
20		EP300	15%	c.A3377G	p.N1126S	nonsynonymous SNV	no
20		PIK3CA	55%	c.A3140G	p.H1047R	nonsynonymous SNV	yes
20		NOTCH1	24%	c.A5137G	p.N1713D	nonsynonymous SNV	no
20		MAP3K4	61%	c.81_82insCCG	p.P27delinsPP	nonframeshift insertion	no
20	Metastasis	USH2A	56%	c.C11881G	p.L3961V	nonsynonymous SNV	no
20		PTEN	44%	c.C284T	p.P95L	nonsynonymous SNV	no
20		SACS	5%	c.G12346T	p.D4116Y	nonsynonymous SNV	no
20		ERCC4	49%	c.T2551G	p.F851V	nonsynonymous SNV	no
20		ERBB2	7%	c.T2264C	p.L755S	nonsynonymous SNV	yes
20		ARHGAP35	ND	c.T3284G	p.V1095G	nonsynonymous SNV	no
20		EP300	54%	c.A3377G	p.N1126S	nonsynonymous SNV	no
20		PIK3CA	14%	c.A3140G	p.H1047R	nonsynonymous SNV	yes
20		SMO	42%	c.C2233A	p.Q745K	nonsynonymous SNV	no
20		FANCC	26%	c.G1378A	p.G460S	nonsynonymous SNV	no
20		NOTCH1	8%	c.A5137G	p.N1713D	nonsynonymous SNV	no
20		MYCN	62%	c.473_512del	p.158_171del	frameshift deletion	no
20		GNAS	6%	c.1113_1148del	p.371_383del	nonframeshift deletion	no
20		MAP3K4	8%	c.81_82insCCG	p.P27delinsPP	nonframeshift insertion	no
21	Primary Tumor	MUTYH	7%	c.C1502T	p.S501F	nonsynonymous SNV	no
21		TSC2	40%	c.G4084T	p.A1362S	nonsynonymous SNV	no
21		MSH6	38%	c.G2501A	p.S834N	nonsynonymous SNV	no

21		SLC4A5	40%	c.A2414G	p.H805R	nonsynonymous SNV	no
21		PIK3CA	61%	c.G1633A	p.E545K	nonsynonymous SNV	yes
21		B2M	39%	c.30_31del	p.10_11del	frameshift deletion	no
21	Metastasis	MUTYH	46%	c.C1502T	p.S501F	nonsynonymous SNV	no
21		TSC2	48%	c.G4084T	p.A1362S	nonsynonymous SNV	no
21		MSH6	5%	c.G2501A	p.S834N	nonsynonymous SNV	no
21		SLC4A5	43%	c.A2414G	p.H805R	nonsynonymous SNV	no
21		PIK3CA	40%	c.G1633A	p.E545K	nonsynonymous SNV	yes
22	Primary Tumor	PIK3CD	22%	c.C1439T	p.P480L	nonsynonymous SNV	no
22		SDHB	47%	c.T487C	p.S163P	nonsynonymous SNV	no
22		NTRK1	4%	c.G2321A	p.R774Q	nonsynonymous SNV	no
22		AIP	3%	c.G47A	p.R16H	nonsynonymous SNV	no
22		SLX4	3%	c.G4034A	p.R1345H	nonsynonymous SNV	no
22		AXIN2	46%	c.G592C	p.E198Q	nonsynonymous SNV	no
22		AXIN2	38%	c.G386C	p.R129P	nonsynonymous SNV	no
22		AXIN2	41%	c.G357C	p.Q119H	nonsynonymous SNV	no
22		ASXL2	61%	c.A2200G	p.T734A	nonsynonymous SNV	no
22		TTLL9	55%	c.T641C	p.I214T	nonsynonymous SNV	no
22		MYB	49%	c.T328C	p.Y110H	nonsynonymous SNV	no
22		GATA3	39%	c.1283_1284insCCTGTCCTTTGGACCACACCACCCCTCCAGCATGGTCA	p.S428fs	frameshift insertion	no
22		ZFHX3	53%	c.3398_3399insCCCCCCCCCCCCACCCCC	p.P1133delinsPPPPPPP	nonframeshift insertion	no
22		BARD1	ND	c.1075_1095del	p.359_365del	nonframeshift deletion	no
22	Metastasis	PIK3CD	19%	c.C1439T	p.P480L	nonsynonymous SNV	no
22		SDHB	45%	c.T487C	p.S163P	nonsynonymous SNV	no
22		NTRK1	4%	c.G2321A	p.R774Q	nonsynonymous SNV	no
22		AIP	4%	c.G47A	p.R16H	nonsynonymous SNV	no

22		SLX4	4%	c.G4034A	p.R1345H	nonsynonymous SNV	no
22		PPM1D	27%	c.C257A	p.P86Q	nonsynonymous SNV	no
22		AXIN2	15%	c.G592C	p.E198Q	nonsynonymous SNV	no
22		AXIN2	36%	c.G386C	p.R129P	nonsynonymous SNV	no
22		AXIN2	60%	c.G357C	p.Q119H	nonsynonymous SNV	no
22		ASXL2	42%	c.A2200G	p.T734A	nonsynonymous SNV	no
22		TTLL9	55%	c.T641C	p.I214T	nonsynonymous SNV	no
22		MYB	14%	c.T328C	p.Y110H	nonsynonymous SNV	no
22		RECQL4	60%	c.G2967T	p.M989I	nonsynonymous SNV	no
22		GATA3	41%	c.1283_1284insCCTGTCCTTTGGACCACACCACCCCTCCAGCATGGTCA	p.S428fs	frameshift insertion	no
22		BARD1	47%	c.1075_1095del	p.359_365del	nonframeshift deletion	no
23	Primary Tumor	MUTYH	18%	c.C1502T	p.S501F	nonsynonymous SNV	no
23		USH2A	48%	c.G1432C	p.E478Q	nonsynonymous SNV	no
23		TP53	45%	c.C152G	p.S51X	stopgain SNV	yes
23		JAK3	91%	c.G2164A	p.V722I	nonsynonymous SNV	no
23		ALK	76%	c.G1079A	p.R360K	nonsynonymous SNV	no
23		MSH6	86%	c.C1508G	p.S503C	nonsynonymous SNV	no
23		PIK3R1	8%	c.G589C	p.D197H	nonsynonymous SNV	no
23		TEK	86%	c.C2824T	p.L942F	nonsynonymous SNV	no
23		PTEN	46%	c.591_593del	p.197_198del	nonframeshift deletion	no
23	Metastasis	MUTYH	44%	c.C1502T	p.S501F	nonsynonymous SNV	no
23		USH2A	40%	c.G1432C	p.E478Q	nonsynonymous SNV	no
23		TP53	35%	c.C152G	p.S51X	stopgain SNV	yes
23		JAK3	53%	c.G2164A	p.V722I	nonsynonymous SNV	no
23		MSH6	4%	c.C1508G	p.S503C	nonsynonymous SNV	no
23		PIK3R1	8%	c.G589C	p.D197H	nonsynonymous SNV	no

23		LTK	4%	c.1210_1211del	p.404_404del	frameshift deletion	no
24	Primary Tumor	PIK3CD	52%	c.G1379A	p.R460H	nonsynonymous SNV	no
24		NTRK1	53%	c.G2321A	p.R774Q	nonsynonymous SNV	no
24		GATA3	27%	c.T881A	p.M294K	nonsynonymous SNV	no
24		ANK3	45%	c.G412A	p.G138R	nonsynonymous SNV	no
24		LTK	40%	c.G1054A	p.V352I	nonsynonymous SNV	no
24		MGA	21%	c.C7051G	p.Q2351E	nonsynonymous SNV	no
24		PALB2	29%	c.G2794A	p.V932M	nonsynonymous SNV	no
24		ERBB4	29%	c.G667A	p.G223R	nonsynonymous SNV	no
24		PIK3CA	10%	c.G1633A	p.E545K	nonsynonymous SNV	yes
24		MAP3K1	53%	c.C2243G	p.S748X	stopgain SNV	no
24		NOTCH4	40%	c.A1363G	p.T455A	nonsynonymous SNV	no
24		GPNMB	30%	c.C950A	p.A317D	nonsynonymous SNV	no
24		MLL3	9%	c.A2922T	p.L974F	nonsynonymous SNV	no
24		MAP3K1	50%	c.1138_1145del	p.380_382del	frameshift deletion	no
24	Metastasis	PIK3CD	56%	c.G1379A	p.R460H	nonsynonymous SNV	no
24		NTRK1	17%	c.G2321A	p.R774Q	nonsynonymous SNV	no
24		GATA3	49%	c.T881A	p.M294K	nonsynonymous SNV	no
24		ANK3	38%	c.G412A	p.G138R	nonsynonymous SNV	no
24		LTK	26%	c.G1054A	p.V352I	nonsynonymous SNV	no
24		MGA	44%	c.C7051G	p.Q2351E	nonsynonymous SNV	no
24		PALB2	24%	c.G2794A	p.V932M	nonsynonymous SNV	no
24		MUC16	39%	c.A19771G	p.N6591D	nonsynonymous SNV	no
24		ERBB4	38%	c.C3536G	p.P1179R	nonsynonymous SNV	no
24		ERBB4	13%	c.G667A	p.G223R	nonsynonymous SNV	no
24		PIK3CA	42%	c.G1633A	p.E545K	nonsynonymous SNV	yes
24		MAP3K1	54%	c.C2243G	p.S748X	stopgain SNV	no

24		NOTCH4	28%	c.A1363G	p.T455A	nonsynonymous SNV	no
24		GPNMB	30%	c.C950A	p.A317D	nonsynonymous SNV	no
24		MLL3	25%	c.A2922T	p.L974F	nonsynonymous SNV	no
24		MAP3K1	42%	c.1138_1145del	p.380_382del	frameshift deletion	no
26	Primary Tumor	TP53	56%	c.A466C	p.N156H	nonsynonymous SNV	no
26		ERCC2	50%	c.C874G	p.Q292E	nonsynonymous SNV	no
26		ITGAV	31%	c.C2074T	p.R692C	nonsynonymous SNV	no
26		ROS1	47%	c.G6607A	p.D2203N	nonsynonymous SNV	no
26		FANCB	47%	c.C1177T	p.P393S	nonsynonymous SNV	no
26		PALB2	16%	c.1882_1890del	p.628_630del	nonframeshift deletion	no
26		TP53	16%	c.484delG	p.E162fs	frameshift deletion	no
26	Metastasis	ERCC2	48%	c.C874G	p.Q292E	nonsynonymous SNV	no
26		ITGAV	21%	c.C2074T	p.R692C	nonsynonymous SNV	no
26		ROS1	49%	c.G6607A	p.D2203N	nonsynonymous SNV	no
		PALB2	41%	c.1882_1890del	p.628_630del	nonframeshift deletion	no

Supplementary Table 3: Targeted therapies selected according to the molecular profiles of primary tumors and metastases according to guidelines of two clinical trials of personalized

Patients	SHIVA trial				MOST trial			
	Primary tumors		Metastases		Primary tumors		Metastases	
	**Drugs**	**Actionable targets**	**Drugs**	**Actionable targets**	**Drugs**	**Actionable targets**	**Drugs**	**Actionable targets**
1	everolimus	*PIK3CA,* NM_006218:c. A3140T:p.H1047L (Hotspot)	everolimus	*PIK3CA,* NM_006218:c. A3140T:p.H1047L (Hotspot)	everolimus	*PIK3CA,* NM_006218:c. A3140T:p.H1047L (Hotspot)	everolimus	*PIK3CA,* NM_006218:c. A3140T:p.H1047L (Hotspot)
2	---	---	---	---	nilotinib	*KIT*, NM_000222:c. T2771C:p. I924T,KIT:NM_001093772:c. T2759C:p.I920T (Damaging)	nilotinib	*KIT*, NM_000222:c.T2771C:p. I924T,KIT:NM_001093772:c. T2759C:p.I920T (Damaging)
4	everolimus	*PIK3CA,* NM_006218:c. A3140G:p.H1047R (Hotspot)	everolimus	*PIK3CA,* NM_006218:c. A3140G:p.H1047R (Hotspot)	everolimus	*PIK3CA,* NM_006218:c. A3140G:p.H1047R (Hotspot)	everolimus	*PIK3CA,* NM_006218:c. A3140G:p.H1047R (Hotspot)
5	everolimus	*PIK3CA,* NM_006218:c. A3140G:p.H1047R (Hotspot)	everolimus	*PIK3CA,* NM_006218:c. A3140G:p.H1047R (Hotspot)	everolimus	*PIK3CA,* NM_006218:c. A3140G:p.H1047R (Hotspot)	everolimus	*PIK3CA,* NM_006218:c. A3140G:p.H1047R (Hotspot)
6	lapatinib+trastuzumab	*ERBB2*, focal amplification	lapatinib+ trastuzumab	*ERBB2*, focal amplification	lapatinib	*ERBB2*, focal amplification	lapatinib	*ERBB2*, focal amplification
7	---	---	---	---	---	---	---	---
8	everolimus	*PIK3CA,* NM_006218:c. G1633A:p.E545K (Hotspot)	---	---	everolimus	*PIK3CA,* NM_006218:c. G1633A:p.E545K (Hotspot)	---	---
9	everolimus	*PIK3CA,* NM_006218:c. G353A:p.G118D (Hotspot)	everolimus	*PIK3CA,* NM_006218:c. G2176A:p.E726K (Hotspot)	everolimus	*PIK3CA,* NM_006218:c. G353A:p.G118D (Hotspot)	everolimus	*PIK3CA,* NM_006218:c. G2176A:p.E726K (Hotspot)
10	everolimus	*AKT1*, NM_001014431:c. G130A:p.D44N (Hotspot)	everolimus	*AKT1*, NM_001014431:c. G130A:p.D44N (Hotspot)	everolimus	*AKT1*, NM_001014431:c. G130A:p.D44N (Hotspot)	everolimus	*AKT1*, NM_001014431:c. G130A:p.D44N (Hotspot)
11	---	---	---	---	---	---	---	---

12	everolimus	*PIK3CA,* NM_006218:c. G1624A:p.E542K (Hotspot)	everolimus	*PIK3CA,* NM_006218:c. G1624A:p.E542K (Hotspot)	everolimus	*PIK3CA,* NM_006218:c. G1624A:p.E542K (Hotspot)	everolimus	*PIK3CA,* NM_006218:c. G1624A:p.E542K (Hotspot)
13	everolimus	*PIK3CA,* NM_006218:c. A3140G:p.H1047R (Hotspot)	everolimus	*PIK3CA,* NM_006218:c. A3140G:p.H1047R (Hotspot)	everolimus	*PIK3CA,* NM_006218:c. A3140G:p.H1047R (Hotspot)	everolimus	*PIK3CA,* NM_006218:c. A3140G:p.H1047R (Hotspot)
14	---	---	---	---	---	---	---	---
15	---	---	---	---	---	---	---	---
16	everolimus	*PIK3CA,* NM_006218:c. C1616G:p.P539R (Hotspot)	everolimus	*PIK3CA,* NM_006218:c. C1616G:p.P539R (Hotspot)	everolimus	*PIK3CA,* NM_006218:c. C1616G:p.P539R (Hotspot)	everolimus	*PIK3CA,* NM_006218:c. C1616G:p.P539R (Hotspot)
17	everolimus	*AKT1*, NM_001014431:c. G49A:p.E17K (Hotspot)	everolimus	*AKT1*, NM_001014431:c. G49A:p.E17K (Hotspot)	everolimus	*AKT1*, NM_001014431:c. G49A:p.E17K (Hotspot)	everolimus	*AKT1*, NM_001014431:c. G49A:p.E17K (Hotspot)
18	everolimus	*PIK3CA,* NM_006218:c. A3140T:p.H1047L (Hotspot)	everolimus	*PIK3CA,* NM_006218:c. A3140T:p.H1047L (Hotspot)	everolimus	*PIK3CA,* NM_006218:c. A3140T:p.H1047L (Hotspot)	everolimus	*PIK3CA,* NM_006218:c. A3140T:p.H1047L (Hotspot)
20	everolimus	*PIK3CA,* NM_006218:c. A3140T:p.H1047L (Hotspot)	everolimus	*PIK3CA,* NM_006218:c. A3140T:p.H1047L (Hotspot)	everolimus	*PIK3CA,* NM_006218:c. A3140T:p.H1047L (Hotspot)	everolimus	*PIK3CA,* NM_006218:c. A3140T:p.H1047L (Hotspot)
21	everolimus	*PIK3CA,* NM_006218:c. G1633A:p.E545K (Hotspot)	everolimus	*PIK3CA,* NM_006218:c. G1633A:p.E545K (Hotspot)	everolimus	*PIK3CA,* NM_006218:c. G1633A:p.E545K (Hotspot)	everolimus	*PIK3CA,* NM_006218:c. G1633A:p.E545K (Hotspot)
22	---	---	---	---	---	---	---	---
23	---	---	---	---	everolimus	*PIK3R1*, NM_001242466:c. G589C:p.D197H (Damaging)	everolimus	*PIK3R1*, NM_001242466:c. G589C:p.D197H (Damaging)
24	everolimus	*PIK3CA,* NM_006218:c. G1633A:p.E545K (Hotspot)	everolimus	*PIK3CA,* NM_006218:c. G1633A:p.E545K (Hotspot)	everolimus	*PIK3CA,* NM_006218:c. G1633A:p.E545K (Hotspot)	everolimus	*PIK3CA,* NM_006218:c. G1633A:p.E545K (Hotspot)
26	lapatinib+trastuzumab	*ERBB2*, focal amplification	lapatinib+ trastuzumab	*ERBB2*, focal amplification	lapatinib	*ERBB2*, focal amplification	lapatinib	*ERBB2*, focal amplification

Supplementary Table S4: **List of 365 genes assessed by targeted NGS**

Symbol	Name	Chromosomal location	Entrez Gene ID
ABL1	c-abl oncogene 1, non-receptor tyrosine kinase	9q34.1	25
ABL2	v-abl Abelson murine leukemia viral oncogene homolog 2	1q25.2	27
ACVR1B	activin A receptor, type IB	12q13	91
ADNP	activity-dependent neuroprotector homeobox	20q13.13	23394
AFF2	AF4/FMR2 family, member 2	Xq28	2334
AIP	aryl hydrocarbon receptor interacting protein	11q13.3	9049
AJUBA	ajuba LIM protein	14q11.2	84962
AKT1	v-akt murine thymoma viral oncogene homolog 1	14q32.32	207
AKT2	v-akt murine thymoma viral oncogene homolog 2	19q13.1-q13.2	208
AKT3	v-akt murine thymoma viral oncogene homolog 3 (protein kinase B, gamma)	1q44	10000
ALK	anaplastic lymphoma receptor tyrosine kinase	2p23	238
ALKBH6	alkB, alkylation repair homolog 6 (E. coli)	19q13.12	84964
ANGPT2	angiopoietin 2	8p23.1	285
ANK3	ankyrin 3, node of Ranvier (ankyrin G)	10q21	288
APC	adenomatous polyposis coli	5q21-q22	324
APOB	apolipoprotein B (including Ag(x) antigen)	2p24-p23	338
AR	androgen receptor	Xq12	367
ARHGAP35	Rho GTPase activating protein 35	19q13.3	2909
ARID1A	AT rich interactive domain 1A (SWI-like)	1p35.3	8289
ARID1B	AT rich interactive domain 1B (SWI1-like)	6q25.1	57492
ARID2	AT rich interactive domain 2 (ARID, RFX-like)	12q12	196528
ARID5B	AT rich interactive domain 5B (MRF1-like)	10q21.2	84159
ASXL2	additional sex combs like 2 (Drosophila)	2p24.1	55252
ATM	ataxia telangiectasia mutated	11q22-q23	472
ATRX	alpha thalassemia/mental retardation syndrome X-linked	Xq21.1	546
AXIN2	axin 2	17q23-q24	8313
AXL	AXL receptor tyrosine kinase	19q13.1	558
B2M	beta-2-microglobulin	15q21-q22.2	567
BAP1	BRCA1 associated protein-1 (ubiquitin carboxy-terminal hydrolase)	3p21.31-p21.2	8314
BARD1	BRCA1 associated RING domain 1	2q34-q35	580
BCLAF1	BCL2-associated transcription factor 1	6q22-q23	9774
BCOR	BCL6 corepressor	Xp11.4	54880
BLK	B lymphoid tyrosine kinase	8p23-p22	640
BLM	Bloom syndrome, RecQ helicase-like	15q26.1	641
BMPR1A	bone morphogenetic protein receptor, type IA	10q22.3	657

BRAF	v-raf murine sarcoma viral oncogene homolog B1	7q34	673
BRCA1	breast cancer 1, early onset	17q21	672
BRCA2	breast cancer 2, early onset	13q12.3	675
BRIP1	BRCA1 interacting protein C-terminal helicase 1	17q22.2	83990
BUB1B	budding uninhibited by benzimidazoles 1 homolog beta (yeast)	15q15	701
C3ORF70	chromosome 3 open reading frame 70	3q27.2	285382
CAP2	CAP, adenylate cyclase-associated protein, 2 (yeast)	6p22.3	10486
CASP8	caspase 8, apoptosis-related cysteine peptidase	2q33-q34	841
CBFB	core-binding factor, beta subunit	16q22.1	865
CCDC6	coiled-coil domain containing 6	10q21	8030
CCL2	chemokine (C-C motif) ligand 2	17q11.2-q12	6347
CCND1	cyclin D1	11q13	595
CCND3	cyclin D3	6p21	896
CCR4	chemokine (C-C motif) receptor 4	3p24	1233
CD22	CD22 molecule	19q13.1	933
CD248	CD248 molecule, endosialin	11q13	57124
CD33	CD33 molecule	19q13.3	945
CD38	CD38 molecule	4p15	952
CD3E	CD3e molecule, epsilon (CD3-TCR complex)	11q23	916
CD3G	CD3g molecule, gamma (CD3-TCR complex)	11q23	917
CD4	CD4 molecule	12p13.31	920
CD40	CD40 molecule, TNF receptor superfamily member 5	20q12-q13.2	958
CD52	CD52 molecule	1p36	1043
CD70	CD70 molecule	19p13	970
CD74	CD74 molecule, major histocompatibility complex, class II invariant chain	5q32	972
CDC27	cell division cycle 27 homolog (S. cerevisiae)	17q21.32	996
CDC73	cell division cycle 73, Paf1/RNA polymerase II complex component, homolog (S. cerevisiae)	1q25	79577
CDH1	cadherin 1, type 1, E-cadherin (epithelial)	16q22.1	999
CDK4	cyclin-dependent kinase 4	12q14	1019
CDKN1B	cyclin-dependent kinase inhibitor 1B (p27, Kip1)	12p13.1-p12	1027
CDKN1C	cyclin-dependent kinase inhibitor 1C (p57, Kip2)	11p15.5	1028
CDKN2A	cyclin-dependent kinase inhibitor 2A	9p21	1029
CDKN2B	cyclin-dependent kinase inhibitor 2B (p15, inhibits CDK4)	9p21	1030
CEBPA	CCAAT/enhancer binding protein (C/EBP), alpha	19q13.1	1050
CEP57	centrosomal protein 57kDa	11q21	9702
CHD4	chromodomain helicase DNA binding protein 4	12p13	1108

CHEK2	checkpoint kinase 2	22q12.1	11200
CREBBP	CREB binding protein	16p13.3	1387
CSF1R	colony stimulating factor 1 receptor	5q32	1436
CTCF	CCCTC-binding factor (zinc finger protein)	16q21-q22.3	10664
CTGF	connective tissue growth factor	6q23.1	1490
CTLA4	cytotoxic T-lymphocyte-associated protein 4	2q33	1493
CTNNB1	catenin (cadherin-associated protein), beta 1, 88kDa	3p21	1499
CUL3	cullin 3	2q36.2	8452
CUX1	cut-like homeobox 1	7q22.1	1523
CYLD	cylindromatosis (turban tumor syndrome)	16q12.1	1540
DDB2	damage-specific DNA binding protein 2, 48kDa	11p12-p11	1643
DDR1	discoidin domain receptor tyrosine kinase 1	6p21.3	780
DDR2	discoidin domain receptor tyrosine kinase 2	1q23.3	4921
DIAPH1	diaphanous homolog 1 (Drosophila)	5q31	1729
DICER1	dicer 1, ribonuclease type III	14q32.13	23405
DIS3L2	DIS3 mitotic control homolog (S. cerevisiae)-like 2	2q37.1	129563
DLL4	delta-like 4 (Drosophila)	15q14	54567
DNER	delta/notch-like EGF repeat containing	2q36.3	92737
EGFL7	EGF-like-domain, multiple 7	9q34.3	51162
EGFR	epidermal growth factor receptor	7p12	1956
EIF2S2	eukaryotic translation initiation factor 2, subunit 2 beta, 38kDa	20q11.2	8894
ELF3	E74-like factor 3 (ets domain transcription factor, epithelial-specific)	1q32.2	1999
EP300	E1A binding protein p300	22q13.2	2033
EPCAM	epithelial cell adhesion molecule	2p21	4072
EPHA2	EPH receptor A2	1p36	1969
EPHB2	EPH receptor B2	1p36.1-p35	2048
ERBB2	v-erb-b2 erythroblastic leukemia viral oncogene homolog 2, neuro/glioblastoma derived oncogene homolog (avian)	17q21.1	2064
ERBB3	v-erb-b2 erythroblastic leukemia viral oncogene homolog 3 (avian)	12q13	2065
ERBB4	v-erb-a erythroblastic leukemia viral oncogene homolog 4 (avian)	2q33.3-q34	2066
ERCC2	excision repair cross-complementing rodent repair deficiency, complementation group 2	19q13.3	2068
ERCC3	excision repair cross-complementing rodent repair deficiency, complementation group 3	2q21	2071
ERCC4	excision repair cross-complementing rodent repair deficiency, complementation group 4	16p13.12	2072

ERCC5	excision repair cross-complementing rodent repair deficiency, complementation group 5	13q33	2073
ESR1	estrogen receptor 1	6q25.1	2099
EXT1	exostosin 1	8q24.11	2131
EXT2	exostosin 2	11p12-p11	2132
EZH2	enhancer of zeste homolog 2 (Drosophila)	7q35-q36	2146
EZR	ezrin	6q25.3	7430
FAM123B	family with sequence similarity 123B	Xq11.2	139285
FANCA	Fanconi anemia, complementation group A	16q24.3	2175
FANCB	Fanconi anemia, complementation group B	Xp22.2	2187
FANCC	Fanconi anemia, complementation group C	9q22.3	2176
FANCD2	Fanconi anemia, complementation group D2	3p26	2177
FANCE	Fanconi anemia, complementation group E	6p22-p21	2178
FANCF	Fanconi anemia, complementation group F	11p15	2188
FANCG	Fanconi anemia, complementation group G	9p13	2189
FANCI	Fanconi anemia, complementation group I	15q26.1	55215
FANCL	Fanconi anemia, complementation group L	2p16.1	55120
FANCM	Fanconi anemia, complementation group M	14q21.2	57697
FAT1	FAT tumor suppressor homolog 1 (Drosophila)	4q35	2195
FBXW7	F-box and WD repeat domain containing 7, E3 ubiquitin protein ligase	4q31.3	55294
FES	feline sarcoma oncogene	15q26.1	2242
FGFR1	fibroblast growth factor receptor 1	8p12	2260
FGFR2	fibroblast growth factor receptor 2	10q26	2263
FGFR3	fibroblast growth factor receptor 3	4p16.3	2261
FGFR4	fibroblast growth factor receptor 4	5q35.1-qter	2264
FGR	Gardner-Rasheed feline sarcoma viral (v-fgr) oncogene homolog	1p36.2-p36.1	2268
FH	fumarate hydratase	1q42.1	2271
FLCN	folliculin	17p11.2	201163
FLT1	fms-related tyrosine kinase 1 (vascular endothelial growth factor/vascular permeability factor receptor)	13q12	2321
FLT3	fms-related tyrosine kinase 3	13q12	2322
FLT4	fms-related tyrosine kinase 4	5q35.3	2324
FNTA	farnesyltransferase, CAAX box, alpha	8p11	2339
FOXA1	forkhead box A1	14q12-q13	3169
FOXQ1	forkhead box Q1	6p25	94234
FRK	fyn-related kinase	6q21-q22.3	2444
FUBP1	far upstream element (FUSE) binding protein 1	1p31.1	8880
FYN	FYN oncogene related to SRC, FGR, YES	6q21	2534

FZD7	frizzled family receptor 7	2q33	8324
GATA2	GATA binding protein 2	3q21.3	2624
GATA3	GATA binding protein 3	10p15	2625
GNAS	GNAS complex locus	20q13.3	2778
GNPTAB	N-acetylglucosamine-1-phosphate transferase, alpha and beta subunits	12q23.2	79158
GOT1	glutamic-oxaloacetic transaminase 1, soluble (aspartate aminotransferase 1)	10q24.1-q25.1	2805
GPC3	glypican 3	Xq26.1	2719
GPNMB	glycoprotein (transmembrane) nmb	7p15	10457
HCK	hemopoietic cell kinase	20q11-q12	3055
HGF	hepatocyte growth factor (hepapoietin A; scatter factor)	7q21.1	3082
HLA-A	major histocompatibility complex, class I, A	6p21.3	3105
HLA-B	major histocompatibility complex, class I, B	6p21.3	3106
HLA-DRB5	major histocompatibility complex, class II, DR beta 5	6p21.3	3127
HNF1A	HNF1 homeobox A	12q24.2	6927
HRAS	v-Ha-ras Harvey rat sarcoma viral oncogene homolog	11p15.5	3265
ICAM1	intercellular adhesion molecule 1	19p13.3-p13.2	3383
IDH1	isocitrate dehydrogenase 1 (NADP+), soluble	2q33.3	3417
IDH2	isocitrate dehydrogenase 2 (NADP+), mitochondrial	15q26.1	3418
IGF1R	insulin-like growth factor 1 receptor	15q26.3	3480
IL12A	interleukin 12A (natural killer cell stimulatory factor 1, cytotoxic lymphocyte maturation factor 1, p35)	3q25.33-q26	3592
IL12B	interleukin 12B (natural killer cell stimulatory factor 2, cytotoxic lymphocyte maturation factor 2, p40)	5q31.1-q33.1	3593
IL1B	interleukin 1, beta	2q14	3553
IL23A	interleukin 23, alpha subunit p19	12q13.3	51561
IL2RA	interleukin 2 receptor, alpha	10p15-p14	3559
IL2RB	interleukin 2 receptor, beta	22q13.1	3560
IL6R	interleukin 6 receptor	1q21	3570
ING1	inhibitor of growth family, member 1	13q34	3621
INPPL1	inositol polyphosphate phosphatase-like 1	11q13	3636
IPO7	importin 7	11p15.4	10527
IRF6	interferon regulatory factor 6	1q32.3-q41	3664
ITGA4	integrin, alpha 4 (antigen CD49D, alpha 4 subunit of VLA-4 receptor)	2q31.3	3676
ITGAL	integrin, alpha L (antigen CD11A (p180), lymphocyte function-associated antigen 1; alpha polypeptide)	16p11.2	3683
ITGAV	integrin, alpha V	2q31-q32	3685
JAK1	Janus kinase 1	1p32.3-p31.3	3716
JAK2	Janus kinase 2	9p24	3717

JAK3	Janus kinase 3	19p13.1	3718
KDM6A	lysine (K)-specific demethylase 6A	Xp11.2	7403
KDR	kinase insert domain receptor (a type III receptor tyrosine kinase)	4q11-q12	3791
KEAP1	kelch-like ECH-associated protein 1	19p13.2	9817
KIT	v-kit Hardy-Zuckerman 4 feline sarcoma viral oncogene homolog	4q11-q12	3815
KLHL8	kelch-like 8 (Drosophila)	4q22.1	57563
KRAS	v-Ki-ras2 Kirsten rat sarcoma viral oncogene homolog	12p12.1	3845
L3MBTL4	l(3)mbt-like 4 (Drosophila)	18p11.31	91133
LCK	lymphocyte-specific protein tyrosine kinase	1p34.3	3932
LCP1	lymphocyte cytosolic protein 1 (L-plastin)	13q14.3	3936
LTK	leukocyte receptor tyrosine kinase	15q15.1-q21.1	4058
LYN	v-yes-1 Yamaguchi sarcoma viral related oncogene homolog	8q13	4067
MAP2K1	mitogen-activated protein kinase kinase 1	15q22.1-q22.33	5604
MAP2K4	mitogen-activated protein kinase kinase 4	17p12	6416
MAP3K1	mitogen-activated protein kinase kinase kinase 1, E3 ubiquitin protein ligase	5q11.2	4214
MAP3K10	mitogen-activated protein kinase kinase kinase 10	19q13.2	4294
MAP3K13	mitogen-activated protein kinase kinase kinase 13	3q27	9175
MAP3K4	mitogen-activated protein kinase kinase kinase 4	6q26	4216
MAP4K3	mitogen-activated protein kinase kinase kinase kinase 3	2p22.1	8491
MAPK1	mitogen-activated protein kinase 1	22q11.21	5594
MAX	MYC associated factor X	14q23	4149
MBD1	methyl-CpG binding domain protein 1	18q21	4152
MBTPS1	membrane-bound transcription factor peptidase, site 1	16q24	8720
MEN1	multiple endocrine neoplasia I	11q13	4221
MET	met proto-oncogene (hepatocyte growth factor receptor)	7q31	4233
MGA	MAX gene associated	15q14	23269
MICALCL	MICAL C-terminal like	11p15.3	84953
MLH1	mutL homolog 1, colon cancer, nonpolyposis type 2 (E. coli)	3p21.3	4292
MLL	myeloid/lymphoid or mixed-lineage leukemia (trithorax homolog, Drosophila)	11q23	4297
MLL2	myeloid/lymphoid or mixed-lineage leukemia 2	12q13.12	8085
MLL3	myeloid/lymphoid or mixed-lineage leukemia 3	7q36.1	58508
MLLT4	myeloid/lymphoid or mixed-lineage leukemia (trithorax homolog, Drosophila); translocated to, 4	6q27	4301
MORC4	MORC family CW-type zinc finger 4	Xq22.3	79710
MS4A1	membrane-spanning 4-domains, subfamily A, member 1	11q12	931

MSH2	mutS homolog 2, colon cancer, nonpolyposis type 1 (E. coli)	2p21	4436
MSH6	mutS homolog 6 (E. coli)	2p16	2956
MSLN	mesothelin	16p13.3	10232
MST1R	macrophage stimulating 1 receptor (c-met-related tyrosine kinase)	3p21.3	4486
MTOR	mechanistic target of rapamycin (serine/threonine kinase)	1p36.2	2475
MUC1	mucin 1, cell surface associated	1q21	4582
MUC16	mucin 16, cell surface associated	19p13.2	94025
MUTYH	mutY homolog (E. coli)	1p34.1	4595
MYB	v-myb myeloblastosis viral oncogene homolog (avian)	6q22-q23	4602
MYCN	v-myc myelocytomatosis viral related oncogene, neuroblastoma derived (avian)	2p24.3	4613
MYH9	myosin, heavy chain 9, non-muscle	22q13.1	4627
NBN	nibrin	8q21	4683
NBPF1	neuroblastoma breakpoint family, member 1	1p36.13	55672
NCAM1	neural cell adhesion molecule 1	11q23.1	4684
NCOR1	nuclear receptor corepressor 1	17p11.2	9611
NCOR2	nuclear receptor corepressor 2	12q24	9612
NF1	neurofibromin 1	17q11.2	4763
NF2	neurofibromin 2 (merlin)	22q12.2	4771
NFE2L2	nuclear factor (erythroid-derived 2)-like 2	2q31	4780
NGF	nerve growth factor (beta polypeptide)	1p13.1	4803
NOTCH1	notch 1	9q34.3	4851
NOTCH2	notch 2	1p13-p11	4853
NOTCH3	notch 3	19p13.2-p13.1	4854
NOTCH4	notch 4	6p21.3	4855
NR1I2	nuclear receptor subfamily 1, group I, member 2	3q12-q13.3	8856
NRAS	neuroblastoma RAS viral (v-ras) oncogene homolog	1p13.2	4893
NSD1	nuclear receptor binding SET domain protein 1	5q35	64324
NTN4	netrin 4	12q22	59277
NTRK1	neurotrophic tyrosine kinase, receptor, type 1	1q21-q22	4914
NTRK2	neurotrophic tyrosine kinase, receptor, type 2	9q22.1	4915
NTRK3	neurotrophic tyrosine kinase, receptor, type 3	15q25	4916
OTUD7A	OTU domain containing 7A	15q13.3	161725
PALB2	partner and localizer of BRCA2	16p12.2	79728
PARP1	poly (ADP-ribose) polymerase 1	1q41-q42	142
PCBP1	poly(rC) binding protein 1	2p13-p12	5093
PDCD1	programmed cell death 1	2q37.3	5133

PDGFRA	platelet-derived growth factor receptor, alpha polypeptide	4q12	5156
PDGFRB	platelet-derived growth factor receptor, beta polypeptide	5q33.1	5159
PHOX2B	paired-like homeobox 2b	4p12	8929
PIGF	phosphatidylinositol glycan anchor biosynthesis, class F	2p21-p16	5281
PIK3CA	phosphoinositide-3-kinase, catalytic, alpha polypeptide	3q26.3	5290
PIK3CB	phosphoinositide-3-kinase, catalytic, beta polypeptide	3q22.3	5291
PIK3CD	phosphoinositide-3-kinase, catalytic, delta polypeptide	1p36.2	5293
PIK3CG	phosphoinositide-3-kinase, catalytic, gamma polypeptide	7q22.3	5294
PIK3R1	phosphoinositide-3-kinase, regulatory subunit 1 (alpha)	5q13.1	5295
PLK1	polo-like kinase 1	16p12.2	5347
PMS1	PMS1 postmeiotic segregation increased 1 (S. cerevisiae)	2q31.1	5378
PMS2	PMS2 postmeiotic segregation increased 2 (S. cerevisiae)	7p22.2	5395
POLE	polymerase (DNA directed), epsilon, catalytic subunit	12q24.3	5426
PPM1D	protein phosphatase, Mg2+/Mn2+ dependent, 1D	17q23.2	8493
PPP2R1A	protein phosphatase 2, regulatory subunit A, alpha	19q13.41	5518
PPP2R5C	protein phosphatase 2, regulatory subunit B', gamma	14q32.31	5527
PRF1	perforin 1 (pore forming protein)	10q22	5551
PRKAR1A	protein kinase, cAMP-dependent, regulatory, type I, alpha (tissue specific extinguisher 1)	17q23-q24	5573
PRKCB	protein kinase C, beta	16p11.2	5579
PRKCE	protein kinase C, epsilon	2p21	5581
PRKCG	protein kinase C, gamma	19q13.4	5582
PRKCH	protein kinase C, eta	14q23.1	5583
PRKCQ	protein kinase C, theta	10p15	5588
PSMB5	proteasome (prosome, macropain) subunit, beta type, 5	14q11.2	5693
PSMC3IP	PSMC3 interacting protein	17q21.2	29893
PTCH1	patched 1	9q22.3	5727
PTEN	phosphatase and tensin homolog	10q23.3	5728
PTK2	PTK2 protein tyrosine kinase 2	8q24.3	5747
PTPN11	protein tyrosine phosphatase, non-receptor type 11	12q24	5781
PTPRD	protein tyrosine phosphatase, receptor type, D	9p23-p24.3	5789
RAC1	ras-related C3 botulinum toxin substrate 1 (rho family, small GTP binding protein Rac1)	7p22	5879
RAD51C	RAD51 homolog C (S. cerevisiae)	17q25.1	5889
RAD51D	RAD51 paralog D	17q11	5892
RAF1	v-raf-1 murine leukemia viral oncogene homolog 1	3p25	5894
RASA1	RAS p21 protein activator (GTPase activating protein) 1	5q13.3	5921

RB1	retinoblastoma 1	13q14.2	5925
RBM10	RNA binding motif protein 10	Xp11.23	8241
RECQL4	RecQ protein-like 4	8q24.3	9401
RET	ret proto-oncogene	10q11.2	5979
RHBDF2	rhomboid 5 homolog 2 (Drosophila)	17q25.1	79651
RHOA	ras homolog family member A	3p21.3	387
RIT1	Ras-like without CAAX 1	1q22	6016
ROS1	c-ros oncogene 1 , receptor tyrosine kinase	6q22	6098
RPS6KC1	ribosomal protein S6 kinase, 52kDa, polypeptide 1	1q41	26750
RSBN1L	round spermatid basic protein 1-like	7q11.23	222194
RUNX1	runt-related transcription factor 1	21q22.3	861
RUNX3	runt-related transcription factor 3	1p36	864
RXRA	retinoid X receptor, alpha	9q34.3	6256
SACS	spastic ataxia of Charlevoix-Saguenay (sacsin)	13q12	26278
SBDS	Shwachman-Bodian-Diamond syndrome	7q11.21	51119
SDHAF2	succinate dehydrogenase complex assembly factor 2	11q12.2	54949
SDHB	succinate dehydrogenase complex, subunit B, iron sulfur (Ip)	1p36.1-p35	6390
SDHC	succinate dehydrogenase complex, subunit C, integral membrane protein, 15kDa	1q23.3	6391
SDHD	succinate dehydrogenase complex, subunit D, integral membrane protein	11q23	6392
SELP	selectin P (granule membrane protein 140kDa, antigen CD62)	1q22-q25	6403
SF3B1	splicing factor 3b, subunit 1, 155kDa	2q33.1	23451
SGK1	serum/glucocorticoid regulated kinase 1	6q23	6446
SIRT1	sirtuin 1	10q21.3	23411
SIRT4	sirtuin 4	12q	23409
SLAMF7	SLAM family member 7	1q23.1-q24.1	57823
SLC1A3	solute carrier family 1 (glial high affinity glutamate transporter), member 3	5p13	6507
SLC44A3	solute carrier family 44, member 3	1p21.3	126969
SLC4A5	solute carrier family 4, sodium bicarbonate cotransporter, member 5	2p13	57835
SLX4	SLX4 structure-specific endonuclease subunit homolog (S. cerevisiae)	16p13.3	84464
SMAD2	SMAD family member 2	18q21.1	4087
SMAD4	SMAD family member 4	18q21.1	4089
SMARCA4	SWI/SNF related, matrix associated, actin dependent regulator of chromatin, subfamily a, member 4	19p13.2	6597

SMARCB1	SWI/SNF related, matrix associated, actin dependent regulator of chromatin, subfamily b, member 1	22q11.23|22q11	6598
SMO	smoothened, frizzled family receptor	7q32.3	6608
SNX25	sorting nexin 25	4q35.1	83891
SOS1	son of sevenless homolog 1 (Drosophila)	2p21	6654
SOX17	SRY (sex determining region Y)-box 17	8q11.23	64321
SPOP	speckle-type POZ protein	17q21.33	8405
SRC	v-src sarcoma (Schmidt-Ruppin A-2) viral oncogene homolog (avian)	20q12-q13	6714
STAG2	stromal antigen 2	Xq25	10735
STAT5B	signal transducer and activator of transcription 5B	17q11.2	6777
STK11	serine/threonine kinase 11	19p13.3	6794
STX2	syntaxin 2	12q24.33	2054
SUFU	suppressor of fused homolog (Drosophila)	10q24.32	51684
TAP1	transporter 1, ATP-binding cassette, sub-family B (MDR/TAP)	6p21.3	6890
TBC1D1	TBC1 (tre-2/USP6, BUB2, cdc16) domain family, member 1	4p14	23216
TBL1XR1	transducin (beta)-like 1 X-linked receptor 1	3q26.32	79718
TBX3	T-box 3	12q24.1	6926
TCF7L2	transcription factor 7-like 2 (T-cell specific, HMG-box)	10q25.3	6934
TCP11L2	t-complex 11 (mouse)-like 2	12q23.3	255394
TEK	TEK tyrosine kinase, endothelial	9p21	7010
TGFB2	transforming growth factor, beta 2	1q41	7042
TGFB3	transforming growth factor, beta 3	14q24	7043
TGFBR2	transforming growth factor, beta receptor II (70/80kDa)	3p22	7048
TMEM127	transmembrane protein 127	2q11.2	55654
TNFRSF10A	tumor necrosis factor receptor superfamily, member 10a	8p21	8797
TNFRSF10B	tumor necrosis factor receptor superfamily, member 10b	8p22-p21	8795
TNFRSF11A	tumor necrosis factor receptor superfamily, member 11a, NFKB activator	18q22.1	8792
TNFRSF12A	tumor necrosis factor receptor superfamily, member 12A	16p13.3	51330
TNFRSF8	tumor necrosis factor receptor superfamily, member 8	1p36	943
TNFRSF9	tumor necrosis factor receptor superfamily, member 9	1p36	3604
TNFSF13B	tumor necrosis factor (ligand) superfamily, member 13b	13q32-q34	10673
TP53	tumor protein p53	17p13.1	7157
TPX2	TPX2, microtubule-associated, homolog (Xenopus laevis)	20q11.2	22974
TRIM23	tripartite motif containing 23	5q12.3	373
TRPC1	transient receptor potential cation channel, subfamily C, member 1	3q23	7220

TSC1	tuberous sclerosis 1	9q34	7248
TSC2	tuberous sclerosis 2	16p13.3	7249
TTLL9	tubulin tyrosine ligase-like family, member 9	20q11.21	164395
TXNDC8	thioredoxin domain containing 8 (spermatozoa)	9q31.3	255220
U2AF1	U2 small nuclear RNA auxiliary factor 1	21q22.3	7307
USH2A	Usher syndrome 2A (autosomal recessive, mild)	1q41	7399
VEGFA	vascular endothelial growth factor A	6p12	7422
VEGFB	vascular endothelial growth factor B	11q13	7423
VHL	von Hippel-Lindau tumor suppressor, E3 ubiquitin protein ligase	3p25.3	7428
WRN	Werner syndrome, RecQ helicase-like	8p12	7486
WT1	Wilms tumor 1	11p13	7490
XPA	xeroderma pigmentosum, complementation group A	9q22.3	7507
XPC	xeroderma pigmentosum, complementation group C	3p25	7508
YES1	v-yes-1 Yamaguchi sarcoma viral oncogene homolog 1	18p11.31-p11.21	7525
ZFHX3	zinc finger homeobox 3	16q22.3	463
ZHX2	zinc fingers and homeoboxes 2	8q24.13	22882
ZNF471	zinc finger protein 471	19q13.43	57573
ZNF620	zinc finger protein 620	3p22.1	253639
ZNF750	zinc finger protein 750	17q25.3	79755
ZRANB3	zinc finger, RAN-binding domain containing 3	2q21.3	84083
